# Supplementary material for: Phages and their satellites encode hotspots of antiviral systems
Source: Cell Host Microbe. 2022 May 11;30(5):740–753.e5. doi: 10.1016/j.chom.2022.02.018 (PMC9122126; doi:10.1016/j.chom.2022.02.018)
Supplement: Document S1. Figures S1–S7 [file mmc1.pdf]

Cell Host & Microbe, Volume *30*

Supplemental information

Phages and their satellites encode hotspots of antiviral systems

François Rousset, Florence Depardieu, Solange Miele, Julien Dowding, Anne-Laure Laval, Erica Lieberman, Daniel Garry, Eduardo P.C. Rocha, Aude Bernheim, and David Bikard

Supplementary Figures

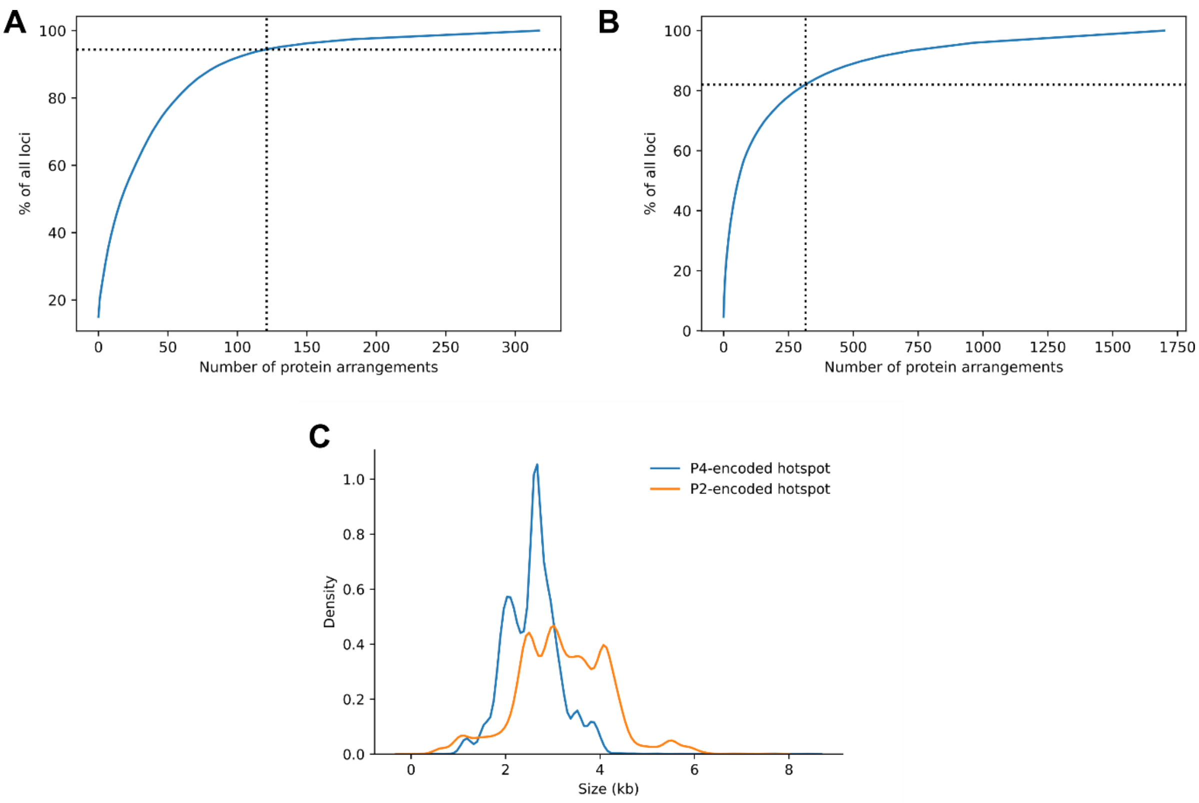

Figure S1. Genomic characteristics of P4 and P2-encoded hotspots, Related to Figure 1 and Figure 4. Plots show the evolution of the frequency of each gene arrangement from P4-encoded (**A**) or P2-encoded (**B**) hotspots. Dashed lines highlight the analyzed threshold: (**A**) 121 arrangements occurring at least 5 times in the P4-encoded hotspot account for 94.4% of all P4-encoded loci, while (**B**) 316 arrangements occurring at least 10 times in the P2-encoded hotspot account for 82% of all P2-encoded loci. (**C**) We measured the size of the P4-encoded hotspot at each locus as the genomic distance between the end of *psu* and the end of *int*. For the P2-encoded hotspot, we used the distance between the end of *A* and the end of *Q*. P2-like phages encode larger systems than P4-like satellites on average (Mann-Whitney p-value < 10^{-100}).

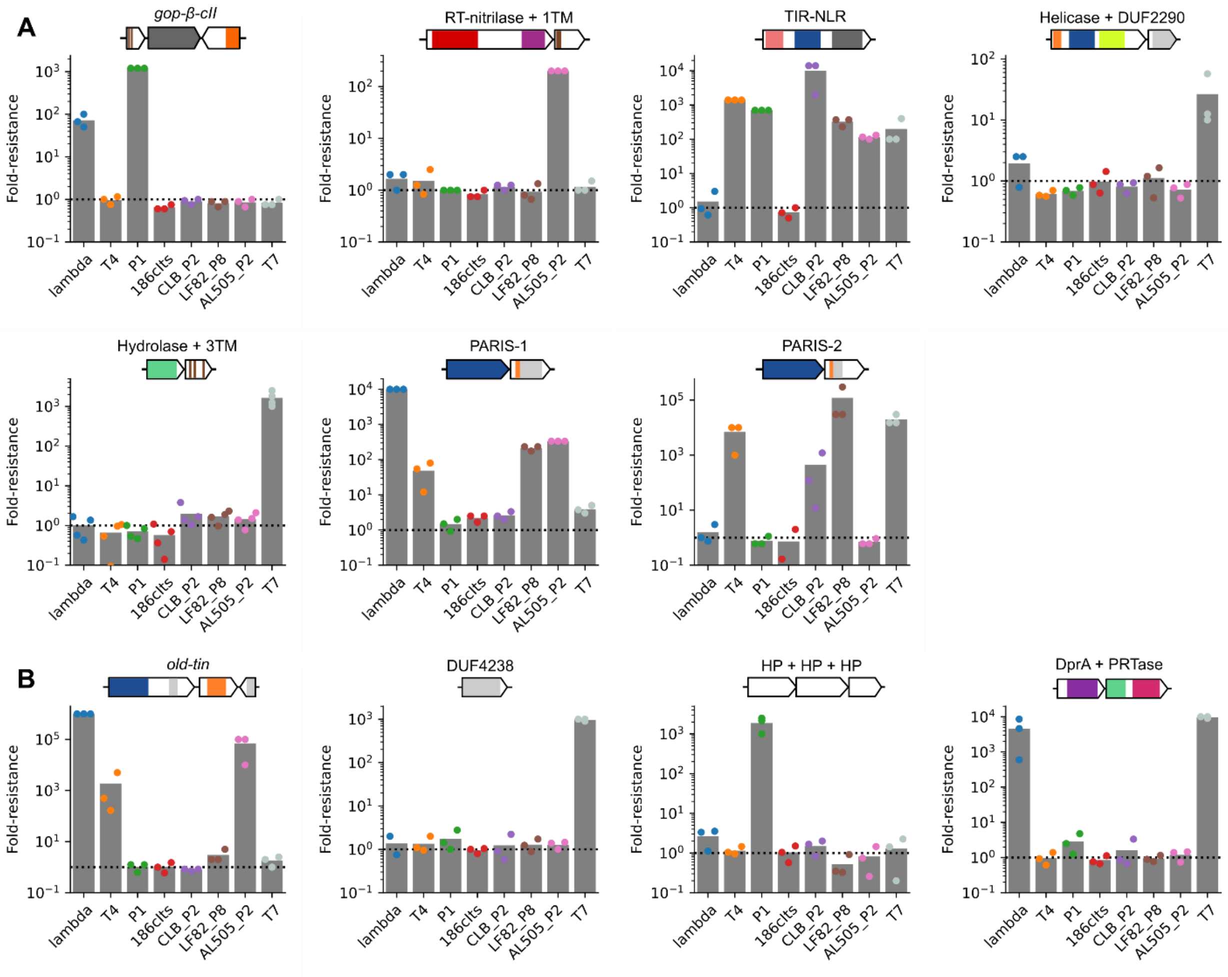

Figure S2. Detailed fold-resistance of all verified defense systems against 8 phages, Related to Figure 2 and Figure 5. Plaque-forming units were measured for each of 8 phages on cells harboring either a control plasmid or a defense system from P4 (**A**) or P2 (**B**) hotspot. Fold-resistance was measured as the ratio between these two values. Bar plots show the mean of 3 to 4 independent measurements. All systems were expressed from their native promoter and measured at 37°C, except the RT-nitrilase + 1TM system that was measured at room temperature and PARIS-1 that was measured in the presence of aTc to induce expression. RT: reverse-transcriptase, TM; transmembrane helix, DUF: domain of unknown function, HP: hypothetical protein, TIR: Toll/Interleukin-1 Receptor, NLR: NOD-like Receptor, PRTase: phosphoribosyltransferase.

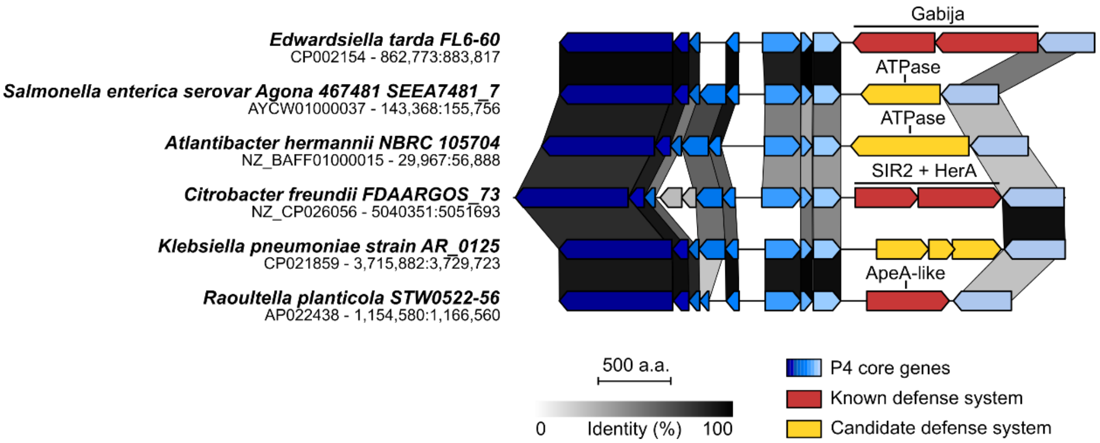

Figure S3. Genetic diversity encoded on P4-like satellites outside *E. coli*, Related to Figure 1. Genomic comparison of P4-like satellites in six different genomes of Enterobacteriaceae. P4 core genes are shown in blue shades. Grey shades show the percentage of identity between homologous proteins from different genomes. Genome accession numbers and positions are shown on the left.

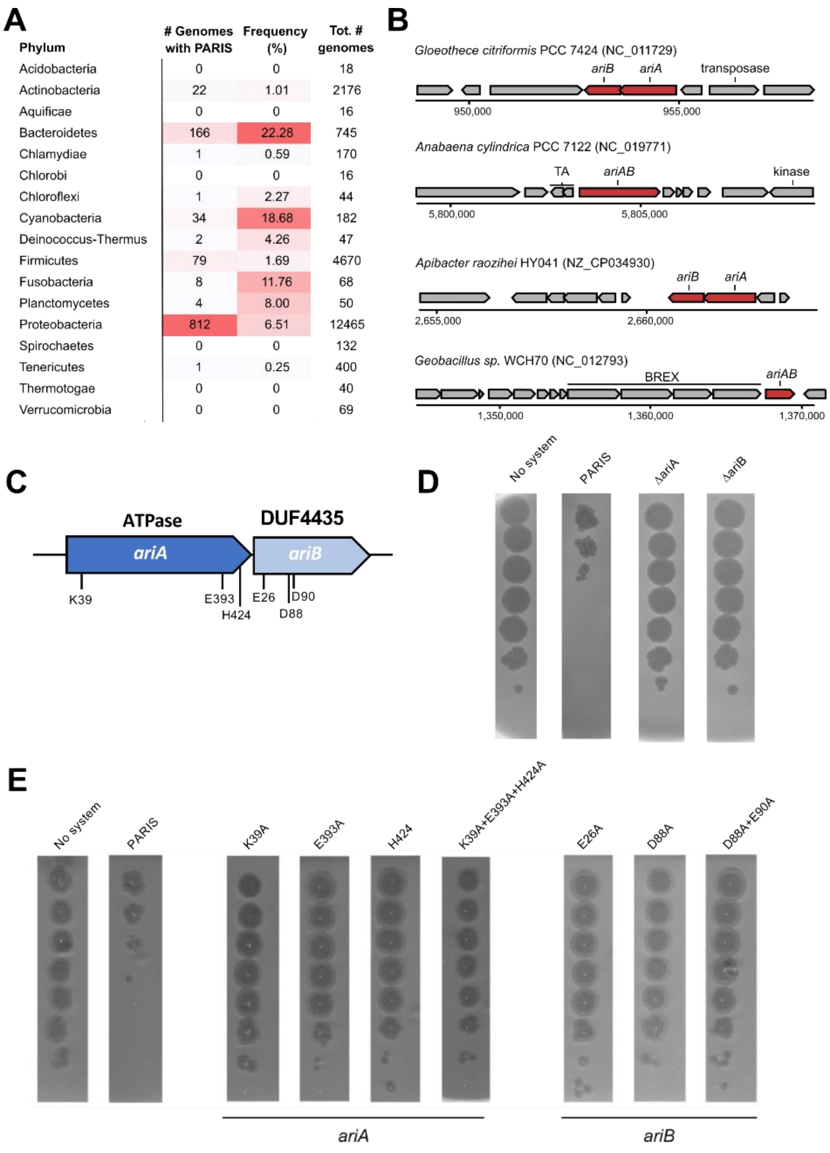

Figure S4. Detection of PARIS in different prokaryotic phyla and defense phenotype of catalytic site mutants. Related to Figure 3. (**A**) PARIS was searched in 21,738 prokaryotic genomes using HMM searches and MacSyFinder (Tesson et al., 2021). For each clade, cells show the number and proportion of genomes where we detected PARIS. A color gradient is used to depict prevalence. (**B**) Genomic view of a few occurrences of PARIS in bacterial genomes. PARIS occurs either as a two-protein system or as a single protein fusion. PARIS genes are colored in red. Genome accessions are shown in parentheses and the bottom track shows genome coordinates. (**C**) Catalytic residues of PARIS in AriA and AriB were predicted using HHpred (Zimmermann et al., 2018). Resistance against phage T7 was measured for each gene deletion (**D**) and single or multiple amino acid mutants (**E**). Plaque assays are representative of three independent replicates. See also Table S7B.

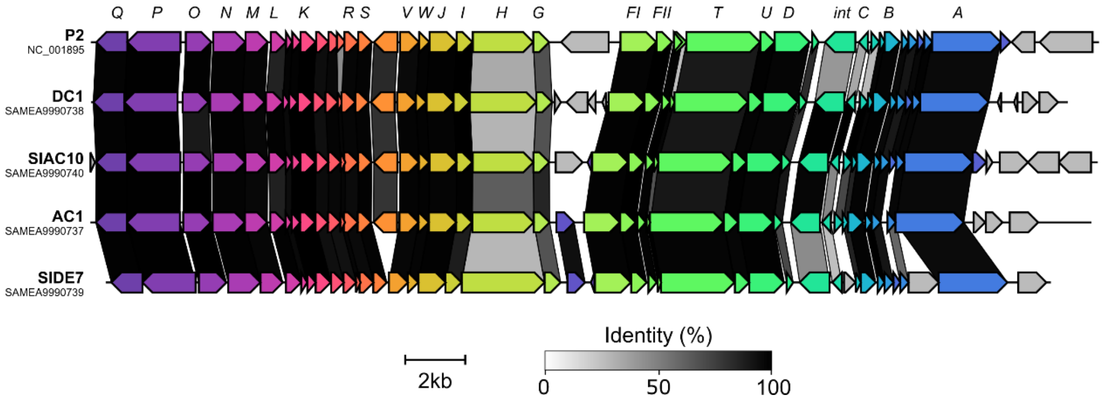

Figure S5. Genomic comparison of canonical phage P2 with newly isolated P2-like phages, Related to Figure 5. Genomes were aligned and visualized using clinker and clustermap.js (Gilchrist and Chooi, 2021). Genes are colored by homologous groups. Grey shades illustrate protein identity.

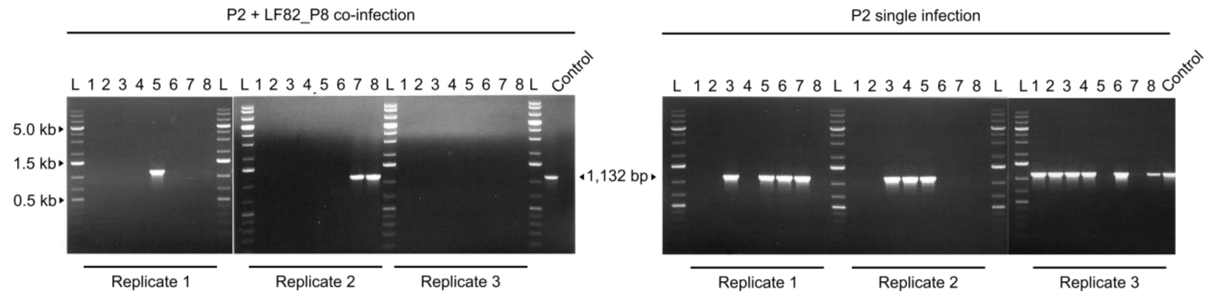

Figure S6. Surviving colonies after co-infection of PARIS-expressing cells by P2 and LF82_P8 include P2 lysogens, Related to Figure 6. E. coli C carrying PARIS (pFD235) and P4-Kan was infected by P2 and LF82_P8 in 1:1 ratio (left) or by P2 alone as a control (right) (see methods). Clones obtained on LB + Cm + Kan plates after infection were screened for the presence of P2 lysogens. A P2-specific PCR was performed on 16 clones for each replicate. The number of positives was 2, 3 and 2, for replicates 1, 2 and 3 respectively after coinfection, and 9, 5 and 13 after infection by P2 alone. The estimated rate of lysogeny is thus ~15% after coinfection and 56% after infection by P2 alone. A P2 lysogen in *E. coli* C was used as a positive control for PCR (see methods). Representative agarose gels for each replicate are shown.

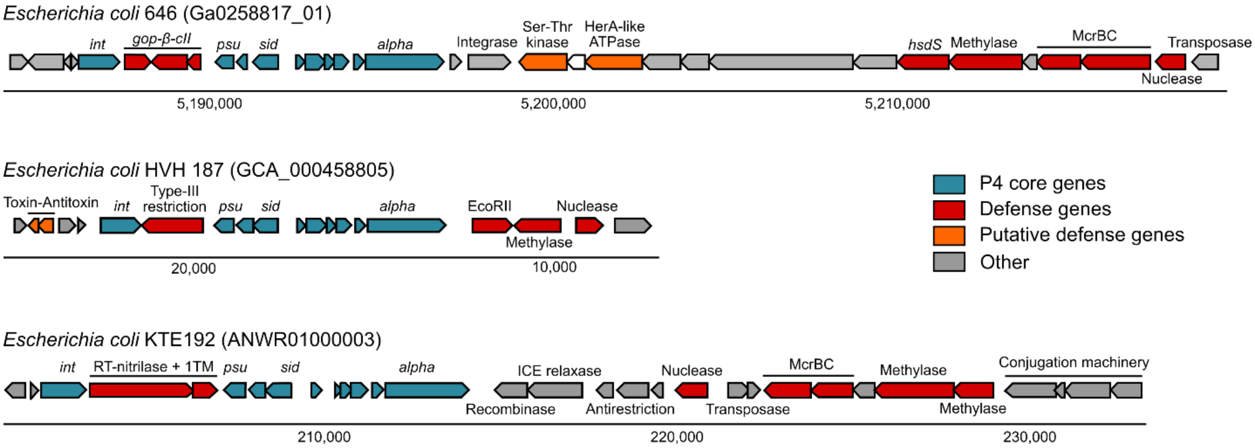

Figure S7. Genomic view of P4-like elements integrated near defense systems and integrative conjugative elements, Related to Figure 1. P4 genes are colored in blue while defense-associated genes are colored in orange or red. Genome accession numbers are shown in parentheses and the bottom tracks shows genomic coordinates.
