# Supplementary material for: Phages and their satellites encode hotspots of antiviral systems
Source: Cell Host Microbe. 2022 May 11;30(5):740–753.e5. doi: 10.1016/j.chom.2022.02.018 (PMC9122126; doi:10.1016/j.chom.2022.02.018)
Supplement: Document S2. Article plus supplemental information [file mmc9.pdf]

Article

Cell Host & Microbe

Phages and their satellites encode hotspots of antiviral systems

Graphical abstract

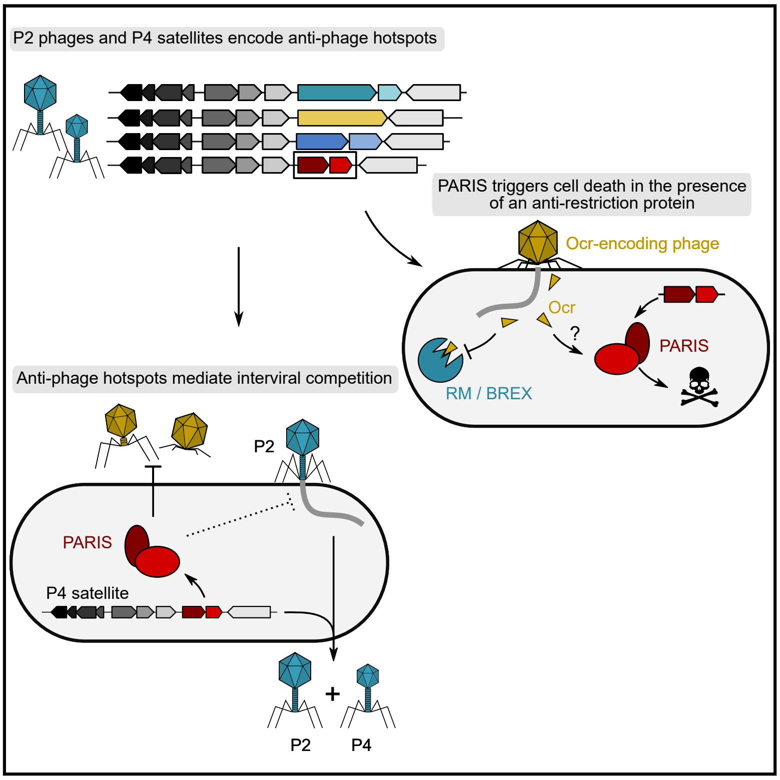

Authors

François Rousset, Florence Depardieu, Solange Miele, ..., Eduardo P.C. Rocha, Aude Bernheim, David Bikard

Correspondence

rousset.fra@gmail.com (F.R.),
david.bikard@pasteur.fr (D.B.)

In brief

Rousset, Depardieu et al. show that *E. coli* P2-like phages and their parasitic P4-like satellites carry diverse anti-phage systems. The PARIS system induces abortive infection, triggered by a phage-encoded anti-restriction protein. Anti-phage systems can turn the parasitic satellite-phage relationship into a mutualistic one when facing a common threat.

Highlights

- P2 phages and P4 satellites carry a genetic hotspot of diverse anti-phage systems
- The PARIS system kills the host in the presence of a phage anti-restriction protein
- Satellites are parasites of phages, but anti-phage systems can make them mutualistic
- Hotspots of anti-phage systems are found in prophages of diverse species

Rousset et al., 2022, Cell Host & Microbe *30*, 740–753
May 11, 2022 © 2022 The Authors. Published by Elsevier Inc.
https://doi.org/10.1016/j.chom.2022.02.018

CellPress

CellPress
OPEN ACCESS

Cell Host & Microbe

Article

Phages and their satellites encode hotspots of antiviral systems

François Rousset,[1,5,6,*] Florence Depardieu,[1,5] Solange Miele,[1] Julien Dowding,[1] Anne-Laure Laval,[1] Erica Lieberman,[2] Daniel Garry,[2] Eduardo P.C. Rocha,[3] Aude Bernheim,[4] and David Bikard[1,7,*]
[1]Institut Pasteur, Université de Paris, CNRS UMR 6047, Synthetic Biology, 75015 Paris, France
[2]Eligo Bioscience, Paris, France
[3]Institut Pasteur, Université de Paris, CNRS UMR 3525, Microbial Evolutionary Genomics, 75015 Paris, France
[4]Université de Paris, INSERM, IAME, 75006 Paris, France
[5]These authors contributed equally
[6]Present address: Department of Molecular Genetics, Weizmann Institute of Science, Rehovot 7610001, Israel
[7]Lead contact
*Correspondence: rousset.fra@gmail.com (F.R.), david.bikard@pasteur.fr (D.B.)
https://doi.org/10.1016/j.chom.2022.02.018

SUMMARY

Bacteria carry diverse genetic systems to defend against viral infection, some of which are found within prophages where they inhibit competing viruses. Phage satellites pose additional pressures on phages by hijacking key viral elements to their own benefit. Here, we show that *E. coli* P2-like phages and their parasitic P4-like satellites carry hotspots of genetic variation containing reservoirs of anti-phage systems. We validate the activity of diverse systems and describe PARIS, an abortive infection system triggered by a phage-encoded anti-restriction protein. Antiviral hotspots participate in inter-viral competition and shape dynamics between the bacterial host, P2-like phages, and P4-like satellites. Notably, the anti-phage activity of satellites can benefit the helper phage during competition with virulent phages, turning a parasitic relationship into a mutualistic one. Anti-phage hotspots are present across distant species and constitute a substantial source of systems that participate in the competition between mobile genetic elements.

INTRODUCTION

Bacteria employ an arsenal of defense strategies to overcome infection by bacteriophages (Bernheim and Sorek, 2020). Recent studies have shown that bacterial immunity is much more diverse that previously envisioned, spanning various mechanisms including DNA restriction (Goldfarb et al., 2015; Kuzmenko et al., 2020; Ofir et al., 2018, 2021; Xiong et al., 2020), abortive infection (Bobonis et al., 2020a; Cohen et al., 2019; Depardieu et al., 2016; Doron et al., 2018; Dy et al., 2014; Lopatina et al., 2020; Millman et al., 2020a; Owen et al., 2021), chemical interference (Bernheim et al., 2021; Kronheim et al., 2018), or nucleotide depletion (Tal et al., 2021a). A series of remarkable discoveries stemmed from the observation that bacterial defense systems tend to cluster in genomic regions termed defense islands (Makarova et al., 2011, 2013). The systematic investigation of genes found in association with known defense genes has considerably expanded our knowledge of bacterial immunity (Doron et al., 2018; Gao et al., 2020). Despite these notable findings, it is believed that many defense systems remain to be discovered. Beyond the description of the defensive arsenal of bacteria, the mechanisms that drive the variability, diversity, and number of defensive functions in bacterial genomes are just beginning to be explored (Tesson et al., 2021).

Recent studies of large ecological datasets of phage-bacteria interactions in *Vibrio* have highlighted how mobile genetic elements (MGEs) such as the SXT integrative conjugative element (ICE) carry anti-phage functions (LeGault et al., 2021). MGEs seem to largely explain the differences in susceptibility of closely related strains to different phages (Hussain et al., 2021). An important source of genetic variability is caused by temperate phages, which have been known for a long time to carry anti-phage functions. During lysogeny, phage survival is tied to host survival, providing a selective pressure for temperate phages to carry "moron" genes that are not essential for the phage but enhance the fitness of their host (Cumby et al., 2012). Prophage-encoded defense systems provide resistance to distant phages through diverse mechanisms, including modification of cell surface receptors (Uc-Mass et al., 2004), inhibition of DNA translocation (McGrath et al., 2002), premature transcription termination (Oberto et al., 1989), or abortive infection (Friedman et al., 2011; Montgomery et al., 2019; Owen et al., 2021; Snyder, 1995). Recent work highlighted how such prophage-encoded defense systems participate in inter-viral competition (Bondy-Denomy et al., 2016; Dedrick et al., 2017; Makarova et al., 2014).

Phages have evolved anti-defense strategies in response to defense systems. The resulting arms race is driving the

 © 2022 The Authors. Published by Elsevier Inc.
This is an open access article under the CC BY license (http://creativecommons.org/licenses/by/4.0/).

diversification and turnover of defense systems in bacteria and counter-defense systems in phages (Bernheim and Sorek, 2020). While bacteria can readily accumulate diverse defense systems in their genome within defense islands, the genome of bacteriophages is typically constrained by the size of the DNA that can be packaged into the capsid, limiting the number of systems they can carry. Genetic diversity in a specific phage clade is typically constrained to specific regions of the phage genome. In P2-like phages from Enterobacteria, two variable loci were described to include anti-phage genes (Nilsson et al., 2004; Odegrip et al., 2006). Anti-defense genes have also been found in variable regions of other phage genomes (Pawluk et al., 2014; Pinilla-Redondo et al., 2020).

Phage satellites represent another important class of MGEs. They highjack the capsid of helper phages to ensure their own propagation and frequently do so while inhibiting the propagation of their helper phage (Fillol-Salom et al., 2020; O'Hara et al., 2017). As such, they are sometimes described as anti-phage systems, but whether satellites could provide defense against non-helper phages remains to be investigated.

Here, we show that hotspots of genetic diversity within the P2-like phage and P4-like satellite families constitute large reservoirs of anti-phage systems. These hotspots are small loci (~1–5 kb) with a high turnover of genetic material located between two conserved genes of the phage or satellite. This is in contrast to defense islands, which consist of chromosomal loci with a high concentration of defense systems and which can span tens of thousands of bases. We describe in more detail phage anti-restriction-induced system (PARIS), an abortive infection system that triggers growth arrest upon sensing an anti-restriction protein. We provide insights into the impact of these hotspots on inter-viral competition and describe diversity hotspots in prophages from distant bacterial species. These findings highlight how prophages constitute a reservoir of defense proteins and point to a strategy to uncover novel anti-phage systems.

RESULTS

P4-like phage satellites encode a hotspot for anti-phage systems

While investigating the determinants of gene essentiality in various *E. coli* strains, we previously identified a reverse-transcriptase (RT) associated with a SLATT-domain protein that is responsible for the essentiality of the exodeoxyribonuclease I SbcB in the *E. coli* strain H120 (Rousset et al., 2021). Since diverse bacterial RTs have recently been implicated in anti-phage defense (Bobonis et al., 2020a, 2020b; Gao et al., 2020; Mestre et al., 2020; Millman et al., 2020a), we investigated the genetic neighborhood of this system. Instead of being in a defense island, it was inserted in a P4-like phage satellite between the polarity suppression protein gene (*psu*) and the integrase (*int*). Since P4-like satellites are very prevalent in Enterobacteria, including 44% of *E. coli* isolates (de Sousa and Rocha, 2022), we inspected other *E. coli* strains and noticed that each P4-like element carried a different genetic system at the same locus (Figure 1A). These elements are adjacent to the *cos* packaging signal and found in either orientation. They include a variety of uncharacterized proteins as well as known defense proteins belonging to type-II and -III restriction-modification (RM) systems and retrons, two of which had previously been reported (Inouye et al., 1991; Kita et al., 1999). The well-characterized P4 satellite (NC_001609) carries at this position the non-essential genes *gop*, β, and *cII*, with *gop* and β likely acting as a toxin-antitoxin pair (Ghisotti et al., 1990). We identified 5,251 occurrences of this locus in >20,000 *E. coli* genomes (STAR Methods; Table S1A;), together encoding >300 different gene arrangements. We analyzed 121 arrangements with at least five occurrences (together accounting for 94.4% of all loci) (Figure S1A) and identified 79 groups of genetic systems (Figure 1B; Table S1B). They can be broadly assigned to three categories: (1) known defense systems such as a type-III restriction enzyme, the type-II RM system EcoO109I (Kita et al., 1999), Kiwa (Doron et al., 2018), Septu (Doron et al., 2018), or SIR2 + HerA (Gao et al., 2020); (2) uncharacterized systems with protein domains that were previously associated with bacterial immunity such as SIR2 (Doron et al., 2018; Gao et al., 2020; Koyuncu et al., 2014), TIR (Ofir et al., 2021), and higher eukaryotes and prokaryotes nucleotide-binding (HEPN) (Anantharaman et al., 2013; Gao et al., 2020); and (3) systems encoding unannotated proteins or whose domain is currently not associated with anti-phage defense, such as haloacid dehydrogenase-like hydrolase (HAD), and various domains of unknown function (DUFs). Based on these observations, we hypothesized that the P4-encoded locus between *psu* and *int* may constitute a reservoir of antiviral systems that likely participate in inter-viral competition.

To test for anti-phage activity, we cloned the set of genes present in the canonical P4 satellite (NC_001609), as well as 18 systems encompassing the three categories above from strains of *Escherichia* and *Klebsiella* under the control of their native promoter on a low copy number vector (Tables S2A and S2B). We introduced them into *E. coli* K-12 MG1655 and challenged each resulting strain with an array of eight coliphages spanning most common phage families (key resources table). When compared with a control vector encoding a green fluorescent protein (GFP), seven systems provided robust and reproducible resistance to at least one phage (Figures 2 and S2; Table S2A).

We report that *gop*-β-*cII* forms an anti-phage system that protects against λ and P1. The *gop* gene product was shown to be toxic in the absence of β (Ghisotti et al., 1990), suggesting that the system may form a toxin-antitoxin system functioning through abortive infection. We also identified a RT from unknown group 5 (Sharifi and Ye, 2021; Toro and Nisa-Martínez, 2014) with a C-terminal nitrilase domain (Table S3) that is associated with a transmembrane effector. This system is reminiscent of a recently described anti-phage system called type-I DRT (Gao et al., 2020), although the RT belongs to a different clade. Validated systems also include a single-gene system with a TIR (toll/interleukin-1-receptor-like) domain protein. TIR domains are important determinants of eukaryotic immunity (Takeda and Akira, 2005) and evidence is emerging of a role for TIR domain proteins as bacterial immune factors as well: the Thoeris defense system carries a TIR protein which produces a second messenger from NAD^+ to activate an associated cell-killing effector (Ofir et al., 2021), while some CBASS and Pycsar systems use a TIR domain to deplete cellular NAD^+ to induce abortive infection (Morehouse et al., 2020; Tal et al., 2021b). Here, the

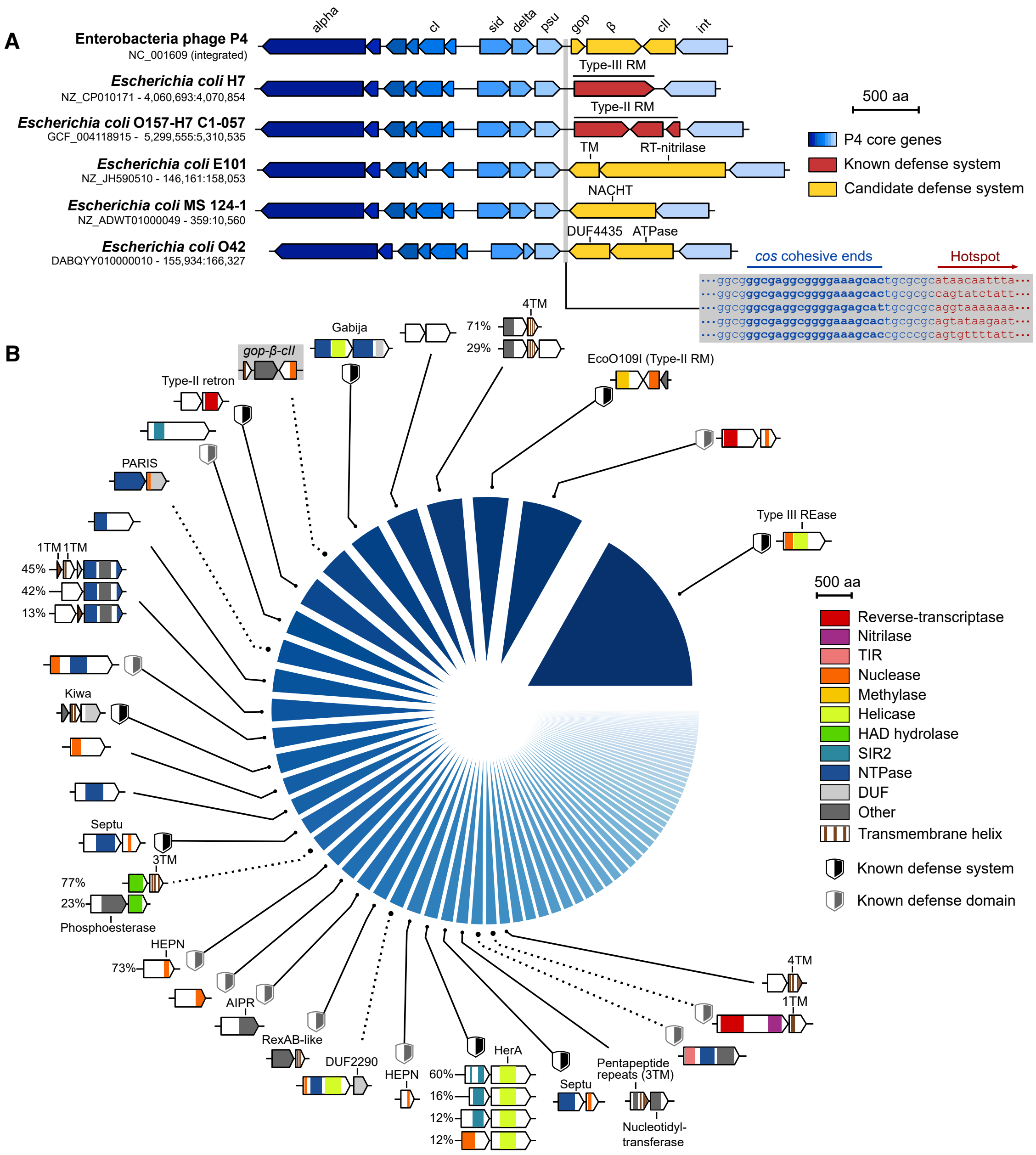

Figure 1. A diversity of genetic systems encoded on P4-like phages in *E. coli*

(A) Genomic visualization of the P4 reference genome and P4-like satellites in five *E. coli* strains, highlighting genetic diversity between *psu* and *int* genes, including known anti-phage systems. Genome accession numbers and positions are shown on the left. The DNA sequence of the *cos*-proximal region in these strains is highlighted with conserved sequences in blue and variable sequences in red.

(B) Systematic analysis of genetic systems encoded between *psu* and *int* identified in 26% (5,251/20,125) of analyzed *E. coli* genomes. The pie chart shows the proportion of loci encoding each of the 30 most abundant systems (Table S1B), shown as gene cassettes colored by protein domains. Validated systems providing phage defense (Figure 2A) are highlighted with a dashed linker. The system from the P4 reference genome is highlighted with a gray background. When a system comprises accessory genes, different variants are shown with the percentage of each occurrence. RT, reverse-transcriptase; REase, restriction-endonuclease; RM, restriction-modification; HEPN, higher eukaryotes and prokaryotes nucleotide-binding; AIPR, abortive infection phage resistance; TIR, Toll/interleukin-1 receptor; HAD, haloacid dehydrogenase-like; SIR2, sirtuin; DUF, domain of unknown function; TM, transmembrane domain.

See also Figures S1 and S3 and Tables S1A and S1B.

TIR protein also harbors C-terminal tetratricopeptide repeats (TPRs) and a central STAND NTPase domain (Leipe et al., 2004) that shares homology with eukaryotic NOD-like receptors (NLRs). In Eukaryotes, the STAND domain is involved in programed cell death and innate immunity by providing ATP/GTP-dependent oligomerization upon signal sensing, leading to downstream signaling (Kufer et al., 2005). In bacteria, STAND NTPases were recently described in the AVAST family of defense

CellPress
OPEN ACCESS

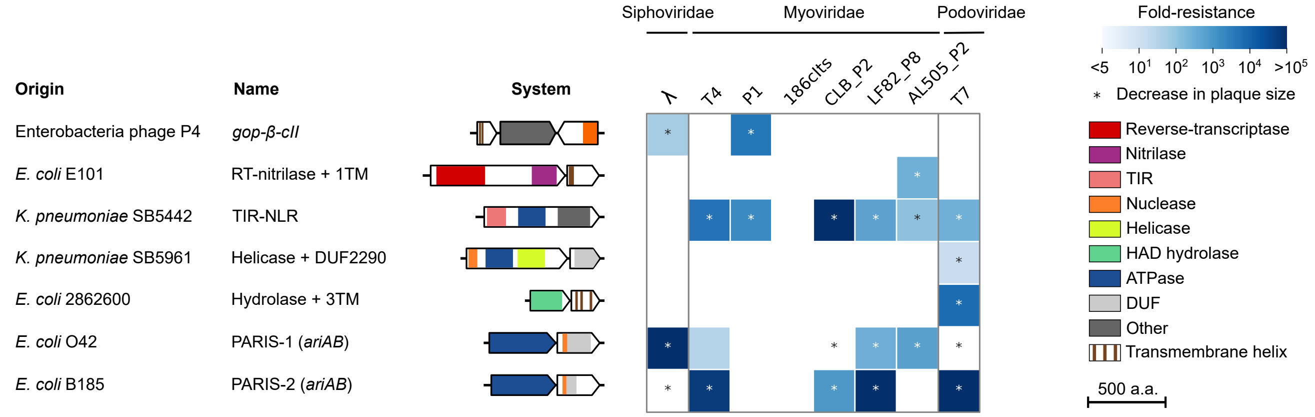

Figure 2. P4-encoded hotspots encode a variety of anti-phage systems

Phage resistance heatmap of the validated defense systems shows the mean fold resistance of three independent replicates against a panel of eight phages (key resources table). Genes are colored by protein family. Genome accession numbers are provided in Table S2A. Systems are under the control of their native promoters, with the exception of PARIS-1, which was only active when expressed from a ptet promoter (pFD237) in the presence of anhydrotetracycline (aTc, 0,5 μg/mL). Defense was measured at 37°C, with the exception of the RT-nitrilase + 1TM system, which was measured at room temperature. RT, reverse-transcriptase; TIR, Toll/interleukin-1 receptor; HAD, haloacid dehydrogenase-like; DUF, domain of unknown function; TM, transmembrane helix.
See also key resources table, Figure S2, and Tables S2A and S3.

systems (Gao et al., 2020). Here, TPRs may sense phage infection, inducing ATP- or GTP-dependent oligomerization and activation of the TIR effector to degrade cellular NAD^+. Other proteins from the hotspot also harbor a STAND NTPase domain coupled to C-terminal repeats, but where the N-terminal TIR effector domain can be replaced by a sirtuin (SIR2) or nuclease (Mrr_cat or BpuSI_N) domain (Table S1B). Taken together, our findings expand the scope of this family of anti-phage systems, which shares striking homology with NLRs from plants and animals.

We also describe two occurrences of an ATPase associated with a DUF4435 protein (described below as PARIS) and two systems that specifically inhibit the growth of phage T7: an ATP-dependent helicase associated with a DUF2290 protein and a HAD-like hydrolase associated with a transmembrane protein. In currently known anti-phage systems, transmembrane proteins are believed to be effectors triggering cell suicide upon infection (Duncan-Lowey et al., 2021; Millman et al., 2020b; Snyder, 1995; Tal et al., 2021b), suggesting that this system could work through abortive infection.

Taken together, our findings show that P4-like satellites carry a hotspot that constitutes a reservoir of anti-phage defense systems. While our bioinformatic search is limited to *E coli* genomes, P4-like elements identified in other Enterobacteriaceae also carry known and candidate defense genes at the same locus (Figure S3).

PARIS triggers growth arrest upon sensing an anti-restriction protein

We further investigated a system comprising an AAA+ ATPase associated with a DUF4435 protein (Figure 2) that we renamed *ariA* and *ariB*, respectively (see below). We detected *ariAB* in 5.2% of bacterial and archaeal genomes from diverse clades (Figures S4A and S4B; Tesson et al., 2021), either as a two-gene cassette as described here or as a single-gene fusion comprising both domains, indicating strong evidence of tight functional interaction (Enright et al., 1999). Sequence analysis of AriB using HHpred identified a ~60-amino acid segment with a weak but significant match (E value = 0.02) to the topoisomerase-primase (TOPRIM) domain of OLD family nucleases (Schiltz et al., 2019, 2020). Interestingly, the association of an ATPase with a TOPRIM domain has previously been found in other defense systems such as P2 *old* that protects against λ (Myung and Calendar, 1995) and GajA from the Gabija system (Cheng et al., 2021; Doron et al., 2018), suggesting that these proteins form a large family of defense proteins. While the system from *E. coli* B185 provided robust defense when expressed from its natural promoter, a distant homolog from *E. coli* O42 initially showed no activity against our phage panel. To ensure that this absence of phenotype was not due to a lack of expression, we cloned this system under the control of an anhydrotetracycline (aTc)-inducible promoter. Upon induction, anti-phage activity was observed for a large range of phages and with a different but overlapping defense profile compared with the system found in *E. coli* B185 (Figure 2).

We then focused on the system from *E. coli* B185 for subsequent experiments. Deletion of either *ariA* or *ariB* was non-toxic and abolished defense, excluding the hypothesis of a toxin-antitoxin system and showing that both components are required for activity (Figures S4C–S4E). Mutations of the predicted ATPase and TOPRIM catalytic residues also abolished defense. We monitored the growth of cells carrying the system or a control plasmid during infection by phage T7 at a low (0.005) or high (5) multiplicity of infection (MOI). In the presence of a control plasmid, infection led to population collapse regardless of the MOI (Figure 3A). In contrast, the system provided partial resistance to T7 at a low MOI but led to a growth halt after infection at a high MOI. In addition, the number of infected cells that released viable phage was reduced by ~15-fold (95% CI: [12.1;18.5]) in the presence of the system, as estimated by a center of infection assay (STAR Methods; Table S4A). Therefore, *ariAB* seem to halt growth in infected cells, preventing the

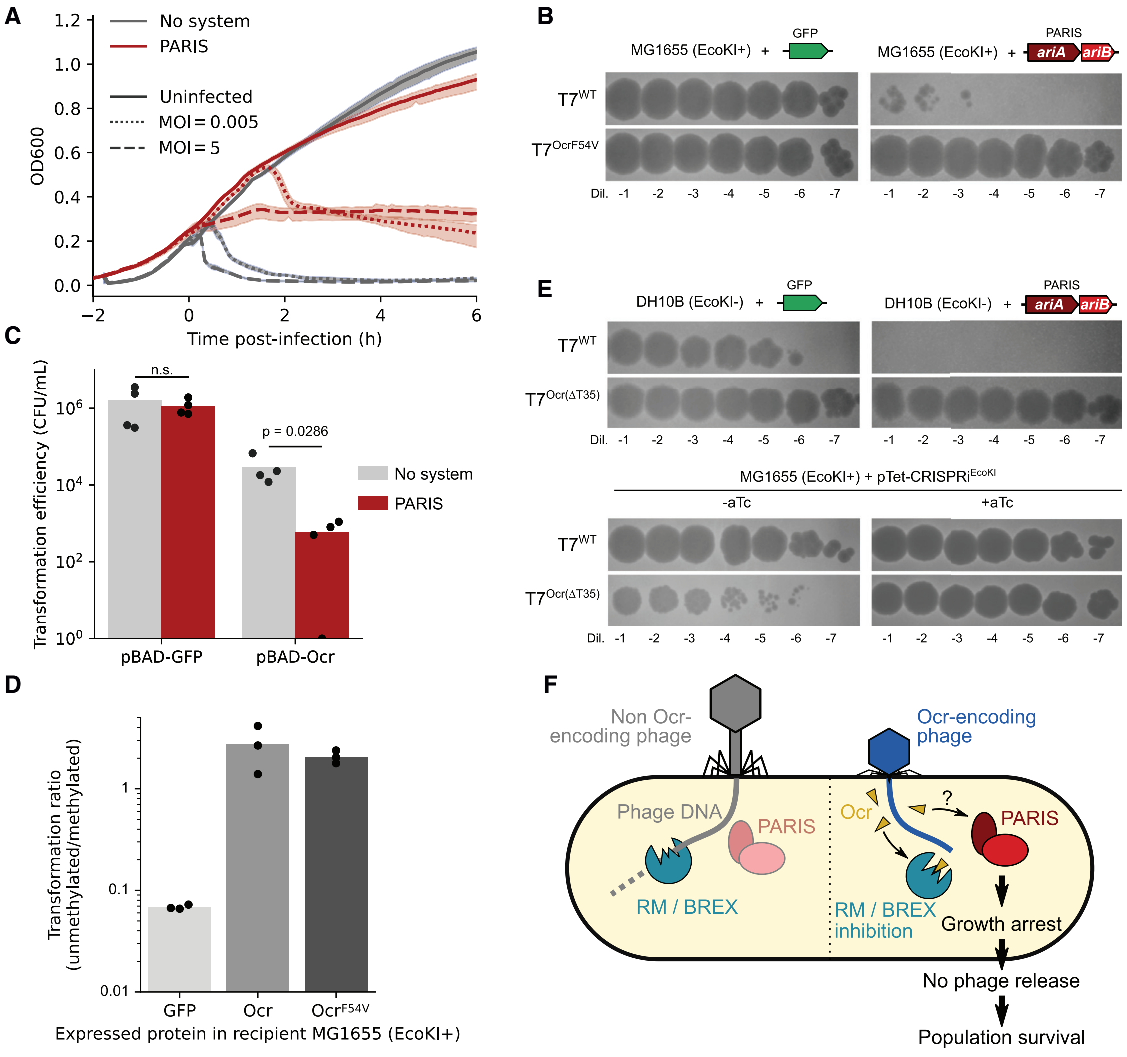

Figure 3. PARIS is triggered by a phage-encoded anti-restriction protein

(A) Time course experiment with cells harboring a control plasmid or a PARIS-encoding plasmid. Cells were kept uninfected or were infected with T7 at a high or low multiplicity of infection (MOI) once cells reached OD ~0.2. Each curve shows the mean of three technical replicates with the standard deviation shown as a transparent area.

(B) Serial dilutions of a high titer lysate of T7 or T7^{OcrF54V} spotted on MG1655.

(C) Transformation efficiency of a plasmid expressing a green fluorescent protein (GFP) or T7 Ocr protein. Bars show the mean of four independent replicates. The p value of a two-sided Mann-Whitney test is shown.

(D) Transformation efficiency of methylated or unmethylated plasmid DNA in MG1655 cells expressing a GFP, wild-type Ocr, or Ocr^{F54V}. Barplot shows the mean of three independent replicates shown as black dots.

(E) Serial dilutions of a high titer lysate of T7or T7$^{Ocr(\Delta T35)}$ spotted on DH10B (top) or on MG1655, expressing an aTc-inducible dCas9 and a sgRNA targeting EcoKI (bottom), representative of three independent replicates.

(F) Current model for the defense activity of PARIS.

See also Figure S4 and Tables S4A and S4B.

completion of the phage cycle, a typical feature of abortive infection systems (Lopatina et al., 2020).

We aimed at identifying the viral trigger by isolating phages that were able to overcome this system, reasoning that mutations in the trigger component would enable phages to escape defense. Whole genome sequencing of four T7 mutants with a restored infectivity (Figure 3B) revealed that all of them carried a mutation (F54V) in gene *0.3*, which encodes Ocr, an anti-restriction protein that inhibits RM and BREX systems by mimicking the structure of DNA (Isaev et al., 2020; Studier, 1975). In the presence of *ariAB*, the transformation efficiency of an Ocr-expressing plasmid was ~100-fold lower than in the absence of the system and yielded only small and sick colonies, while there was no difference when transforming a GFP-encoding plasmid as a control (Figure 3C). This confirmed the toxicity of Ocr in the presence of this system. We can therefore describe it as an anti-anti-restriction system, a remarkable evolutionary strategy where cells have evolved to use a counter-defense

protein as a trigger for abortive infection. This strategy allows cells to undergo self-sacrifice only when an incoming phage can overcome the first line of defense (e.g., a RM or BREX system), therefore maximizing both cellular and population-level survival. As such, we renamed this system PARIS and its components *ariA* and *ariB* (anti-restriction-induced A and B).

During infection by T7, the Ocr protein inhibits EcoKI, the RM system that is naturally present in *E. coli* K-12 MG1655, preventing both restriction and methylation of T7 DNA (Studier, 1975). Therefore, T7 mutants isolated on *E. coli* K-12 MG1655 expressing PARIS must be able to overcome both PARIS and EcoKI. We then wondered if the Ocr^{F54V} mutant that escaped PARIS was still able to block EcoKI. To investigate this, a plasmid carrying an EcoKI restriction motif was extracted either from the strain DH10B (that lacks EcoKI) to yield unmethylated DNA or from strain MG1655 to yield methylated DNA. Transformation efficiency was then measured in MG1655 cells expressing Ocr, Ocr^{F54V}, or a control plasmid. While unmethylated plasmid was clearly restricted by EcoKI, both unmethylated and methylated plasmid DNA could be transformed with similar efficiency when recipient cells expressed Ocr or Ocr^{F54V} (Figure 3D). This shows that Ocr^{F54V} can indeed still block EcoKI.

We then reasoned that the evolutionary pressure on Ocr to maintain EcoKI inhibition should be absent in *EcoKI*- cells. To investigate this, we introduced PARIS in DH10B cells (that lack EcoKI) and selected another set of T7 mutants that escape PARIS. Sequencing the *ocr* gene indeed revealed inactivating frameshifts (ΔT35, ΔG209) or non-sense mutations (247C > T or 265C > T) in 15/16 of isolated mutants (Table S4B). As expected, an *ocr*(ΔT35) mutant of T7 had a reduced efficiency of plaquing on MG1655, but blocking EcoKI expression with dCas9 restored infectivity (Figure 3E). Altogether, our results show that PARIS acts as a second line of defense when a phage is able to inactivate RM systems (Figure 3F), but that anti-restriction proteins can evolve to bypass PARIS while maintaining RM inhibition; e.g., with the F54V mutation in Ocr. A sequence search against the NCBI Viral Database (Hatcher et al., 2017) revealed that the F54 residue is conserved in 93% of Ocr homologs, suggesting that the F54V mutation might have disadvantages in some conditions. Accordingly, a F54A mutation was previously shown to abrogate Ocr dimerization that is required for full inhibition of type-I RM and BREX (Isaev et al., 2020; Zavilgelsky and Kotova, 2014).

A hotspot in P2-like phages encodes diverse anti-phage systems

P4 is a phage satellite that lacks structural genes encoding capsid and tail proteins. Instead, it hijacks capsids from helper phages of the P2 family and uses the P2 terminase to package its own genome into modified P2 capsids (Lindqvist et al., 1993). As a consequence, P4 and P2 share a core *cos* packaging signal (Ziermann and Calendar, 1990). The fact that the defense hotspot is directly adjacent to the *cos* site in P4-like phages prompted us to inspect the *cos*-proximal region in P2-like phages, which is located between the replication protein gpA and the portal protein gpQ (Figure 4A). The P2 reference genome (NC_001895) encodes the accessory phage exclusion genes *old* and *tin* at this locus (Christie and Calendar, 2016). We performed a systematic analysis of this locus in all *E. coli* genomes which showed that this region is another hotspot for genetic diversity as previously suggested (Odegrip et al., 2006), with 1,650 different gene arrangements detected from 18,150 occurrences of this locus (Figure 4B; Table S5A). We curated the arrangements occurring at least 10 times (together accounting for 82% of all loci) (Figure S1B) and identified 169 genetic systems (Table S5B) comprising genes encoding known defense proteins such as retrons and type-III restriction endonucleases, as well as genes likely involved in other functions. For instance, around a fourth of P2-like phages encode a putative plasmid partitioning system that perhaps allows these prophages to be maintained as plasmids, while around 2% encode a cytolethal distending toxin which likely participates in bacterial virulence (Johnson and Lior, 1988). A few systems are common between P4- and P2-encoded hotspots but at different frequencies, while most systems are found in one hotspot but not in the other. Many P2-like hotspots contain more than one system and they tend to be larger than those of the P4-likes (3.2 versus 2.6 kb on average) (Figure S1C), likely reflecting the pressure faced by the latter to package their genome into a capsid of reduced size which can only accommodate ~12 kb (Shore et al., 1978). Accordingly, both phage families encode short systems and lack larger systems such as CRISPR-Cas, BREX, and type-I RM that can be found in bacterial genomes or plasmids.

We cloned eight additional systems from the P2-encoded hotspot and tested their activity against the eight phages described above. In addition, we also cloned the *old* and *tin* genes from bacteriophage P2 to confirm their previously reported anti-phage activity (Christie and Calendar, 2016). The *old-tin* genes provided immunity against a broad range of phages, and we observed defensive activity for three other systems (Figure 5A). The first system consists of a single protein with a DUF4238 domain (whose molecular function is currently unknown) that provides strong resistance against T7. The second system comprises three genes that have no clear predicted domain and protect against P1. Finally, the last system comprises a DNA-processing chain A protein (DprA) associated with a phosphoribosyltransferase (PRTase) and protects against phages T7 and λ. DprA-like proteins are involved in DNA transformation in naturally competent bacteria by binding to single-stranded DNA and interacting with RecA (Mortier-Barrière et al., 2007). Non-competent species also carry DprA-like proteins whose role has remained elusive. Our finding now provides another function to DprA-like proteins in non-competent bacteria. Some PRTase proteins act as probable effectors in bacterial retrons although their mode of action is unknown (Mestre et al., 2020; Millman et al., 2020a). This suggests that DprA could sense phage DNA and activate the PRTase effector, perhaps to trigger cell suicide in an abortive infection mechanism. This hypothesis is supported by the role of DprA homologs as antitoxins in the *shosTA*, *syrTA*, and *rqlHI* toxin-antitoxin systems (Kimelman et al., 2012; Russell and Mulvey, 2015; Sberro et al., 2013). Taken together, our results suggest that both P4 satellites and P2-like phages carry a hotspot of genetic diversity adjacent to the *cos* site, with a large variety of genes involved in anti-phage defense.

We further investigated the ability of P2-like phages to confer protection to their host. We isolated four P2-like phages in *E. coli* C, a restriction-less strain traditionally used in bacteriophage isolation (key resources table; Figure S5; Bertani and Weigle, 1953). We then selected *E. coli* C lysogens and tested their

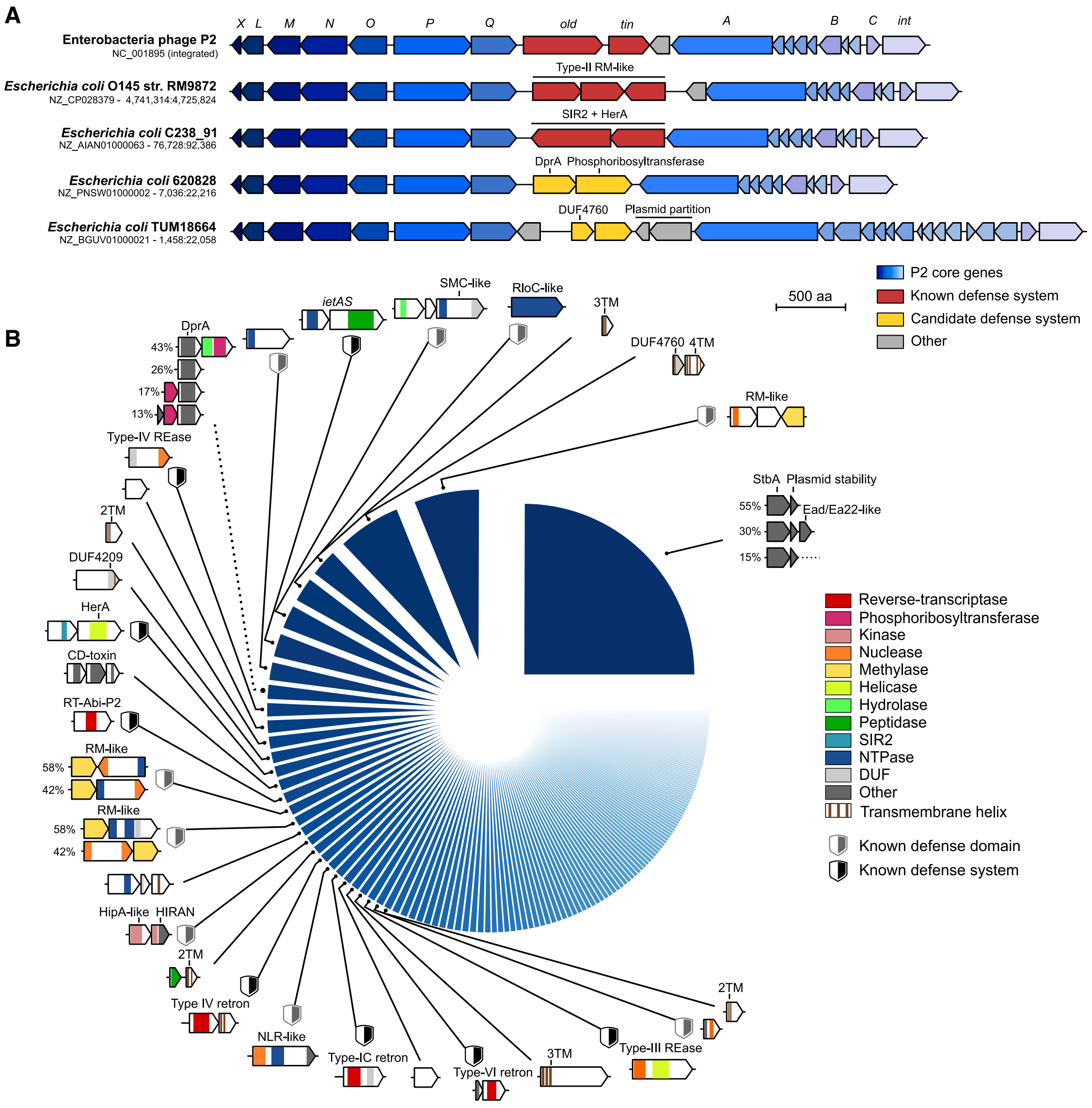

Figure 4. A diversity of genetic systems encoded on P2-like phages in *E. coli*

(A) Visualization of genomic regions from five *E. coli* strains containing a P2-like prophage, highlighting genetic diversity between Q and A genes, including known anti-phage defense systems. Genome accession numbers and positions are shown on the left.

(B) Systematic analysis of genetic systems encoded between gpA and gpQ from P2-like phages. The pie chart shows the 30 most abundant systems classified by prevalence and shown as gene cassettes colored by protein domains (not to scale). When a system comprises accessory genes, different variants are shown, with the percentage of each occurrence on the left. A validated system providing phage defense (Figure 5A) is highlighted with a dashed linker. NLR, NOD-like receptor; SIR2, sirtuin; DUF, domain of unknown function; TM, transmembrane helix; SMC, structural maintenance of chromosome; REase, restriction-endonuclease.

See also Figure S1 and Tables S5A and S5B.

resistance against our phage panel. The P2-like phage AC1 protected its host against λ and LF82_P8 (Figure 5B). At the defense hotspot, AC1 carries a TIR protein that may generate a nucleotide messenger to activate the associated transmembrane proteins (Figure 5C; Ofir et al., 2021). We cloned the three genes present at the AC1 locus on a plasmid that we introduced in *E. coli C* and confirmed their activity against λ and LF82_P8 (Figure 5D). When tested against P2-like phages, this system also provided resistance against P2 itself (Figure 5E), showing that diversity hotspots can participate in inter-viral competition, not only between distant phages but also at a short scale between closely related P2-like phages. Altogether, this confirms that genetic systems carried at the hotspot can provide resistance against diverse phages in their natural context.

Role of defense systems in P2-P4 interactions

The presence of defense systems in P4-like satellites can provide a competitive advantage to a host by providing protection

CellPress
OPEN ACCESS

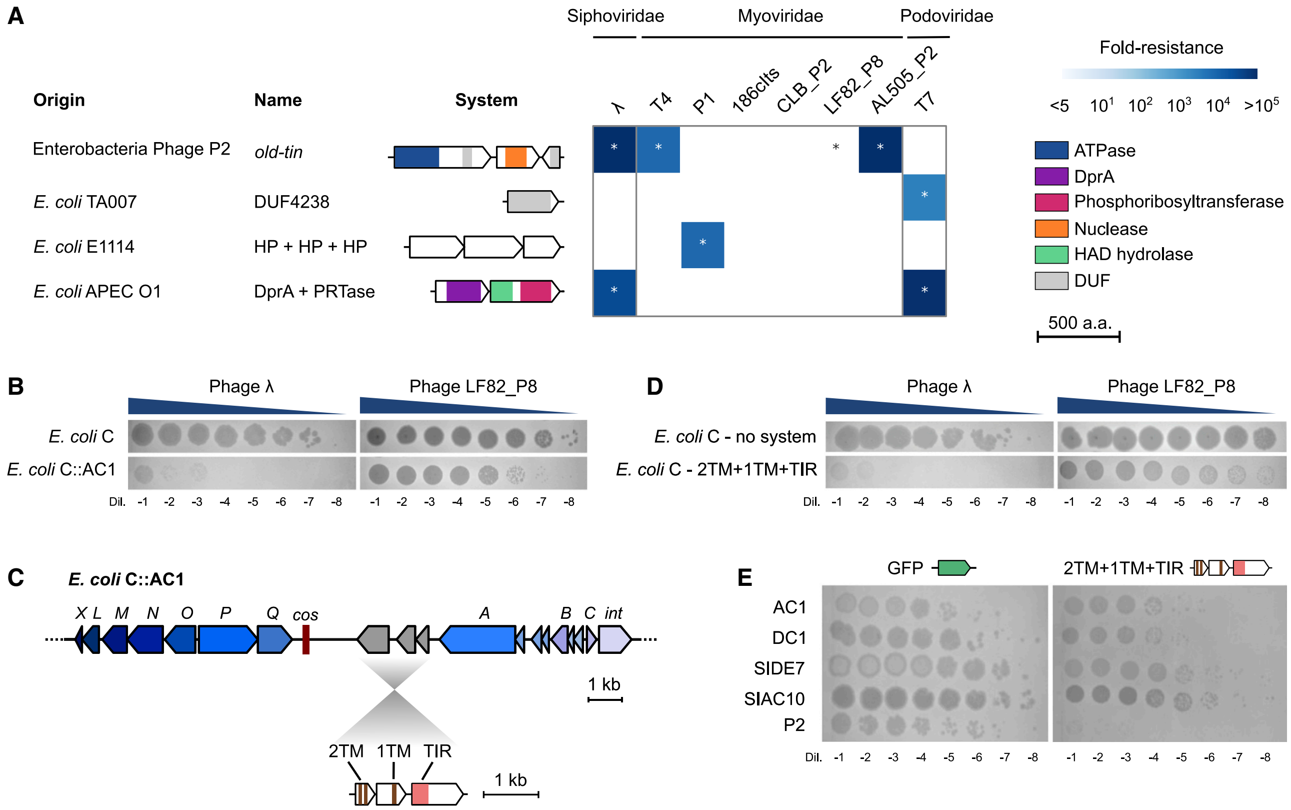

Figure 5. P2-encoded hotspot includes diverse anti-phage systems

(A) Phage resistance heatmaps of the validated defense systems show the median fold resistance of three independent replicates against a panel of eight phages (key resources table). Genes are colored by protein family. Genome accession numbers are provided in Table S2A. HAD, haloacid dehydrogenase-like; HP, hypothetical protein; DUF, domain of unknown function.

(B) Lysogenization of *E. coli* C with P2-like phage AC1 protects against phage λ and LF82_P8.

(C) Description of the system found in P2-like phage AC1. TIR, Toll/interleukin-1 receptor; TM, transmembrane helix.

(D) The candidate defense system from phage AC1 was cloned and introduced into *E. coli* C. The cloned system recapitulates the defense phenotype of the lysogen.

(E) The AC1-encoded system provides protection against P2-like relatives.

See also Figure S5.

against phages. The interests of the bacterium and the phage satellite are however not perfectly aligned. Indeed, when the cell is infected by a phage from the P2 family, P4-like satellites can ensure their own propagation by packaging their DNA into modified P2-like capsids, while the cell dies. We can thus hypothesize that defense systems found in P4-like satellites will usually not restrict the P2-like phages they can hijack or do so while ensuring the propagation of the P4 element. In fact, the only phage in our panel against which no defense could be detected was the P2-like phage 186clts (Figure 2). All the P4-encoded systems described above (Tables S2A and S2B) were introduced into *E. coli* C, and their defense activity was tested against P2 and the four newly isolated P2-like phages. Out of the 18 tested systems, only the TIR-NLR system provided protection (Figure 6A). This is in contrast with the seven systems described in Figure 2 that provided defense against the phages of our panel and the fact that non-P2-like phages were all restricted by several systems. These results suggest that defense systems carried by P4-like satellites are generally permissive to P2-like phages.

We further investigated whether the defense action of the TIR-NLR system against P2-like phages could occur in a timeframe that still enables P4 packaging and transduction. *E. coli* C cells carrying the P420 plasmid, a P4 derivative with a kanamycin resistance gene (Kahn and Helinski, 1978), were infected by P2 in the presence or absence of the TIR-NLR system and P420 titers were measured in the lysate. While P420 particles were still produced in the presence of the defense system, TIR-NLR reduced P420 titers to a similar extent by which it blocked P2 (Figure 6B). This shows that while the TIR-NLR system protects against a broad range of virulent phages, this comes at the cost of limiting the transduction of P4 by P2. Nevertheless, the natural helper phages of TIR-NLR-expressing satellites may not be affected by the system, as is the case for the P2-like phage 186clts (Figure 2).

P4-like satellites can be beneficial to their bacterial host by protecting them from virulent phages. We further hypothesized that by blocking competing virulent phages, a satellite might be beneficial to its helper phage as well. To test this hypothesis, we co-infected *E. coli* C cells carrying P4 with a 1:1 mixture of phages P2 and LF82_P8 in the presence or absence of the

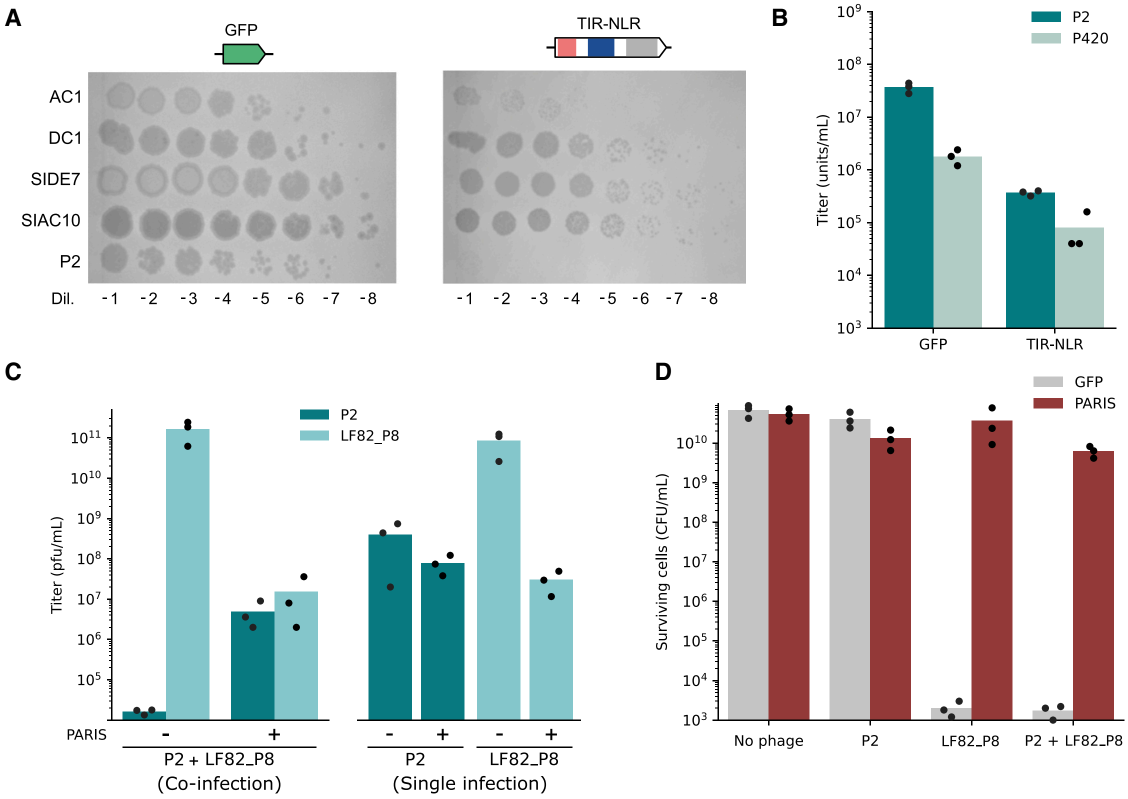

Figure 6. Antiviral hotspots mediate inter-viral competition

(A) Defense activity of the TIR-NLR system against five P2-like phages. Phage dilutions were spotted on a bacterial lawn of *E. coli* C encoding the TIR-NLR system or a GFP control. Representative of three independent replicates.

(B) The TIR-NLR system affects both P2 and P420 propagation. *E. coli* C cells carrying the P420 plasmid, a kanamycin resistant variant of P4 (Kahn and Helinski, 1978), were infected by P2 in the presence of the TIR-NLR system or a GFP control. Titers of P2 and P420 were measured in the lysate (STAR Methods).

(C) PARIS favors P2 during co-infection with LF82_P8. Titers of P2 and LF82_P8 were measured after co-infection at a 1:1 ratio and MOI ~ 0.01 of *E. coli* C cells expressing PARIS or a GFP control (left) (STAR Methods). In parallel, titers of P2 and LF82_P8 were also measured after infection by a single phage (right).

(D) Colony-forming units were measured after single or co-infection by P2 and/or LF82_P8 in *E. coli* C cells expressing PARIS or a GFP control. Bar plots show the mean of three independent replicates, each shown as a black dot.

See also Figure S6.

PARIS system on a plasmid (STAR Methods). In the absence of PARIS, LF82_P8 hindered the propagation of P2 (Figure 6C). In contrast, in the presence of PARIS, LF82_P8 was inhibited and P2 titers were close to those obtained during infection by P2 alone. In addition, PARIS also protected cells from the virulent phage (Figure 6D), thereby favoring lysogeny by P2. We observed ~10^6 more colony-forming units after co-infection in the presence of PARIS than in its absence, among which ~15% were P2 lysogens (Figure S6). These results show how P4 satellites, which are traditionally seen as parasites of P2, might in fact be beneficial to their helper phages by the selective action of their antiviral systems.

Prophage-encoded antiviral hotspots in other species

Finally, we wondered whether defense hotspots also exist in prophages belonging to more distant bacterial species. We were able to identify at least two more occurrences of such hotspots through manual examination of loci that carry some of the systems described here. The first hotspot is prevalent in *Vibrionaceae*, between the cI repressor and the integrase of a P2-related phage that is similar to *Vibrio cholerae* phage K139 (Kapfhammer et al., 2002) and *Aeromonas* phage ΦO18P (Figure 7A; Beilstein and Dreiseikelmann, 2008). The second one is encoded on a prophage from *Bacillus* between a transcriptional regulator and a peptidase adjacent to the integrase (Figure 7B). In both cases, hotspots encode known defense systems such as RM, Septu as well as the Helicase + DUF2290 system, and PARIS described above (Table S6). While most proteins in these loci have currently not been linked to bacterial immunity, many of them have known defense domains, such as nucleases, kinases, and ATPases. This analysis reveals that prophage-encoded diversity hotspots are present in various bacterial organisms and likely constitute a significant reservoir of new antiviral systems across diverse phyla.

DISCUSSION

Our findings showcase a stunning diversity of genetic systems in phages and satellites, with more than 300 unique gene arrangements detected in the P4 hotspot alone. This diversity is matched by the presence of highly diverse defense systems in P2-like phages in the only homologous region between the two types of elements, the *cos* site. Both types of elements include

CellPress
OPEN ACCESS

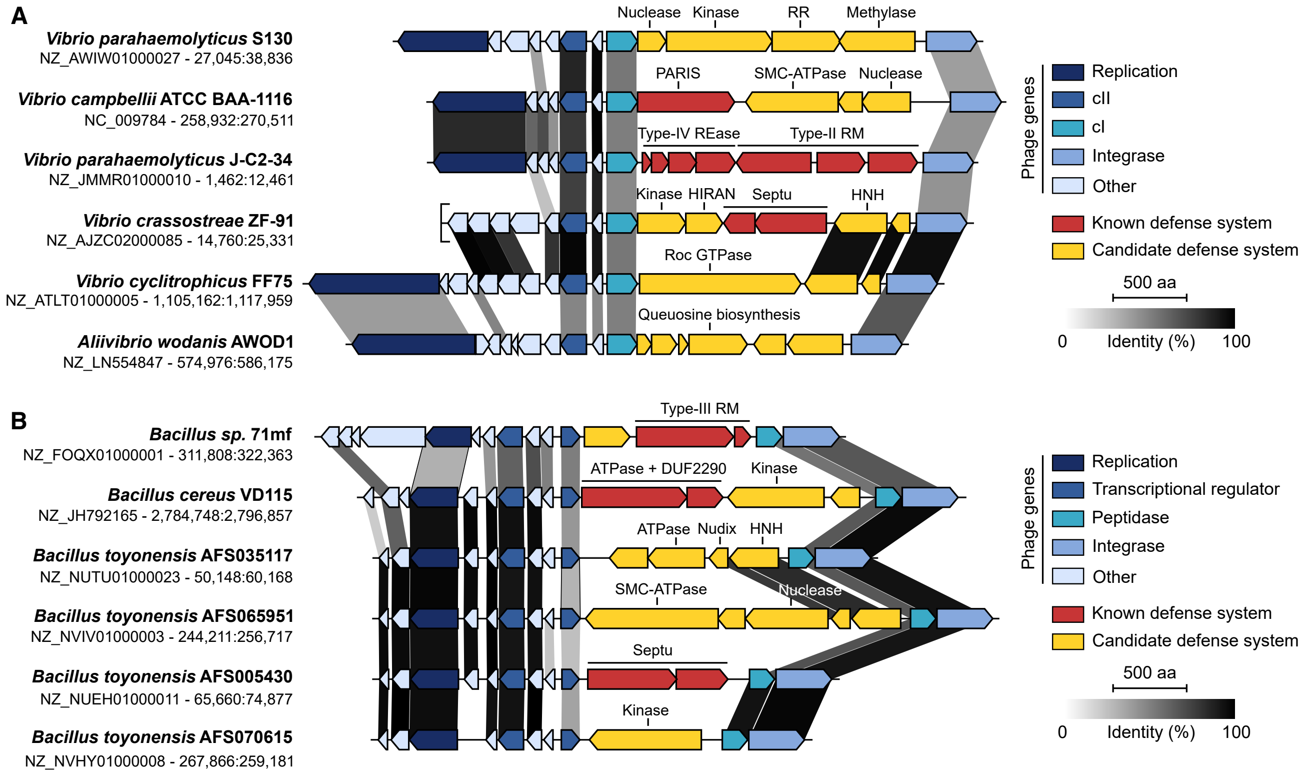

Figure 7. Hotspots for anti-phage systems encoded on other prophage genomes

(A and B) Genomic view of hotspots encoded on prophages from *Vibrionales* (A) and *Bacilliales* (B). Phage genes are shown with different shades of blue. Gray shades show the percentage of identity between homologous proteins from different genomes. Genome accession numbers and positions are shown on the left. See also Table S6.

variants of known systems, or systems carrying combinations of domains previously associated to defense phenotypes, as well as systems with unknown domains or that were not previously associated to defense phenotypes. More than a third of the systems that we cloned showed anti-phage activity against our panel of phages, a proportion that would likely increase by testing more phages. The presence of such a diverse panel of immunity proteins illustrates the extent of inter-viral competition and likely mirrors the diversity of phages encountered by *E. coli* and the anti-defense strategies they deploy.

It is interesting to consider the molecular mechanisms driving the dramatic rate of gene exchange, specifically at these loci. In both P2-like phages and P4-like satellites, the hotspots are directly adjacent to the *cos* sequence. It is tempting to speculate that cohesive ends generated after cleavage by the terminase or made available immediately after injection of the phage DNA into the cytoplasm could provide a substrate for recombination upon co-infection by several P2-like phages or P4-like satellites, or upon co-induction of resident prophages. A second recombination event mediated by homologous recombination would result in genetic exchange of the locus. Although P2- and P4-encoded hotspots generally carry different systems, a few of them are shared, raising the possibility of genetic exchanges between phages and their parasites. Further experimental work will be necessary to validate this or other possible recombination pathways.

Among the many systems, we describe in more detail PARIS, an abortive infection system that senses the T7 Ocr anti-restriction protein (Figure 3). PARIS seems conceptually similar to the anticodon nuclease PrrC, which acts as a second line of defense by sensing the inhibition of the EcoprrI RM system by Stp, a peptide encoded by phage T4 (Penner et al., 1995). Both systems represent a remarkable evolutionary strategy where cells commit suicide only when a first line of defense is compromised, a strategy also recently described in an antiviral retron that senses RecBCD inhibition by a phage protein (Millman et al., 2020a). However, PARIS is distinct in the sense that it does not require the presence of EcoKI or any other type-I RM or BREX system in the cell, as shown by its defensive activity in DH10B cells (Figure 3E). We can thus rule out that PARIS guards EcoKI, but the exact mechanism of anti-restriction sensing by PARIS remains to be unveiled. We found that PARIS also protects against other phages than T7, such as λ and T4, with the two PARIS systems that we assayed offering varying levels of protection (Figure 2). Although the trigger of PARIS remains unknown for those phages, T4 is known to carry the anti-restriction protein Arn, while λ encodes the RecBCD inhibitor Gam, both of which are DNA mimics like Ocr (Court et al., 2007; Ho et al., 2014). Future studies should address whether PARIS is able to sense phage-encoded DNA mimics in general.

The differences in defense profiles provided by different anti-phage systems likely reflects the changing selective pressure

imposed by predation by diverse bacteriophages against which no universal defense exists. Our results point toward a role of P2- and P4-encoded systems in the protection against phages of other families. Defense systems found at the P2 hotspot did not protect against P2-like phages, with the exception of a system from phage AC1, which provided protection against P2 (Figure 5).

Phage satellites were previously described to frequently inhibit their helper phage (Fillol-Salom et al., 2020; O'Hara et al., 2017), but the discovery of defense systems active against a broad range of phages on P4-like satellites raises the question of how they might affect the relationship between the phage, the satellite, and their common host. The existence of abortive infection systems in P4-like elements suggests that it is best for them to kill their host rather than allow an epidemic of phages they cannot highjack. Interestingly, the benefit provided to the host might extend to the helper phage. This is because the selective defense provided by a P4 element favors the propagation of helper P2 phages, either as lysogens or virions, during co-infection with a virulent phage (Figure 6). This challenges the paradigm of the satellite as a phage parasite by showing that in some circumstances the interactions might be mutualistic. The P2 phage propagates the P4 element, while the P4 element gives an advantage to P2 during competition with virulent phages. The diversity of defense systems provided by P4-like elements can then be seen as a rich genetic resource that P2 can harness to protect itself, and its host, against virulent phages without the need to encode all these elements in its own, restricted size, genome.

The integration of temperate phages, their satellites (this work), and ICEs (Johnson et al., 2020; LeGault et al., 2021) carrying defense systems provides a mechanism for the genesis and evolution of defense islands in bacterial genomes. These mobile elements tend to integrate at a small number of chromosomal hotspots (Oliveira et al., 2017). Their inactivation by mutation results in rapid gene loss until only the functions adaptive to the bacterial host remain (Touchon et al., 2014). In fact, we have found occurrences of P4-like satellites that are integrated near defense islands or ICEs that carry defense genes (Figure S7). The high turnover of mobile elements in a few chromosomal hotspots can lead to the accumulation of defense systems in specific loci. Accordingly, recent studies showed how the majority of the flexible genome among close relatives of *Vibrio* consists of anti-phage defense elements (Hussain et al., 2021; Piel et al., 2021). These regions may also be hotbeds for recombination and mutation processes, resulting in novel combinations of protein motifs that may in some cases result in novel defense systems and favoring the genetic entanglement between defense systems, viruses, and MGEs in general (Koonin et al., 2020; Rocha and Bikard, 2022). The bacterial chromosome is much less constrained than those of phages and can thus accumulate many more systems in the same hotspot. In this hypothesis, bacterial defense islands against mobile elements result from the accumulation of defense systems from mobile elements themselves.

Finally, the identification of defense hotspots adjacent to conserved anchor genes provides an alternative strategy to uncover novel defense systems. Unlike previous systematic approaches that rely on the proximity of systems in relatively large loci (Doron et al., 2018; Gao et al., 2020), defense hotspots should be identifiable in metagenomic and virome data with small contigs. We therefore anticipate that this strategy will help uncover more antiviral systems involved in the conflicts between MGEs.

Limitations of the study

We detected the presence of defense hotspots in species other than *E. coli*, but a systematic analysis of the prevalence of such hotspots in phages and satellites across the bacterial kingdom will require a dedicated bioinformatics study that is beyond the scope of this work. In addition, the fraction of defensive systems encoded in the hotspots that we report is likely underestimated due to the small size of our phage panel.

Our findings argue for a role of antiviral hotspots carried by satellites, but more work will be required to ascertain their impact on the eco-evolutionary dynamics of host-phage-satellite interactions in natural *E. coli* hosts. We observed a trend suggesting that defense systems carried by P4 satellites generally do not restrict P2-like phages, but this conclusion is only based on a handful of interactions. The study of more systems and phages will be necessary to draw stronger conclusions. It also remains unclear under what circumstances phage satellites will be selected to block or not block the propagation of their helper phage. More work will also be necessary to establish the fitness benefit that P2 phages can obtain from mobilizing P4 elements and the anti-phage systems they carry. Transduction of P4-like elements by P2-like phages spreads systems that will favor subsequent infection by the latter, but it remains unclear if and when these benefits outweigh the costs paid by the phage in terms of its effective burst size.

Finally, future studies will ascertain how anti-phage systems are captured in defense islands, the role that defense hotspots might play in this process, and the extent of genetic exchanges between phages, satellites, and the host.

STAR★METHODS

Detailed methods are provided in the online version of this paper and include the following:

- KEY RESOURCES TABLE
- RESOURCE AVAILABILITY
  - Lead contact
  - Materials availability
  - Data and code availability
- EXPERIMENTAL MODEL AND SUBJECT DETAILS
  - Bacteria
  - Phages
- METHOD DETAILS
  - Cloning candidate defense systems
  - Phage plaque assays
  - Time course infection experiments
  - Efficiency of centers of infection
  - Isolation and sequencing of mutant phages
  - Transformation assays
  - CRISPRi-mediated EcoKI knockdown
  - Effect of TIR-NLR on P2-P4 interaction
  - P4-Kan construction
  - Competition between P2 and LF82_P8 in the presence of PARIS

- QUANTIFICATION AND STATISTICAL ANALYSIS
  - Identification of prophage-encoded systems
  - Detection of PARIS
  - Identification of hotspots in other bacterial species

SUPPLEMENTAL INFORMATION

Supplemental information can be found online at https://doi.org/10.1016/j.chom.2022.02.018.

ACKNOWLEDGMENTS

We thank Erick Denamur for providing *E. coli* strains and Sylvain Brisse for providing *K. pneumoniae* strains. We also thank Laurent Debarbieux and Mathieu de Jode for providing phages CLB_P2, LF82_P8, and AL505_P2, the P2M platform (Institut Pasteur, Paris, France) for genome sequencing, and Alicia Calvo-Villamañán and Raphaël Laurenceau for their contribution to the PARIS acronym. This work was supported by the European Research Council (ERC) under the Europe Union's Horizon 2020 research and innovation program (grant agreement no. 677823), by the French Government's Investissement d'Avenir program, and by Laboratoire d'Excellence "Integrative Biology of Emerging Infectious Diseases" (ANR-10-LABX-62-IBEID). F.R. is supported by a doctoral scholarship from École normale supérieure. E.P.C.R. was partially supported by the "Fondation pour la Recherche Médicale" (Equipe FRM EQU201903007835).

AUTHOR CONTRIBUTIONS

F.R. and D.B. conceived the project. F.R. and A.B. analyzed the data. F.R., F.D., S.M., A.-L.L., and J.D. performed experiments. E.L. isolated the P2-like phages. D.G. built P4-Kan. F.R., F.D., A.B., E.P.C.R., and D.B. interpreted the results and wrote the manuscript.

DECLARATION OF INTERESTS

D.B. is a founder of Eligo Bioscience and a member of its scientific advisory board.

Received: February 3, 2021
Revised: January 27, 2022
Accepted: February 23, 2022
Published: March 21, 2022

REFERENCES

Abby, S.S., Néron, B., Ménager, H., Touchon, M., and Rocha, E.P.C. (2014). MacSyFinder: a program to mine genomes for molecular systems with an application to CRISPR-Cas systems. PLoS One *9*, e110726.

Anantharaman, V., Makarova, K.S., Burroughs, A.M., Koonin, E.V., and Aravind, L. (2013). Comprehensive analysis of the HEPN superfamily: identification of novel roles in intra-genomic conflicts, defense, pathogenesis and RNA processing. Biol. Direct *8*, 15.

Bankevich, A., Nurk, S., Antipov, D., Gurevich, A.A., Dvorkin, M., Kulikov, A.S., Lesin, V.M., Nikolenko, S.I., Pham, S., Prjibelski, A.D., et al. (2012). SPAdes: a new genome assembly algorithm and its applications to single-cell sequencing. J. Comput. Biol. *19*, 455–477.

Beilstein, F., and Dreiseikelmann, B. (2008). Temperate bacteriophage ΦO18P from an Aeromonas media isolate: characterization and complete genome sequence. Virology *373*, 25–29.

Bernheim, A., Millman, A., Ofir, G., Meitav, G., Avraham, C., Shomar, H., Rosenberg, M.M., Tal, N., Melamed, S., Amitai, G., and Sorek, R. (2021). Prokaryotic viperins produce diverse antiviral molecules. Nature *589*, 120–124.

Bernheim, A., and Sorek, R. (2020). The pan-immune system of bacteria: antiviral defence as a community resource. Nat. Rev. Microbiol. *18*, 113–119.

Bertani, G., and Weigle, J.J. (1953). Host controlled variation in bacterial viruses. J. Bacteriol. *65*, 113–121.

Bobonis, J., Mateus, A., Pfalz, B., Garcia-Santamarina, S., Galardini, M., Kobayashi, C., Stein, F., Savitski, M.M., Elfenbein, J.R., Andrews-Poymenis, H., and Typas, A. (2020b). Bacterial retrons encode tripartite toxin/antitoxin systems. Preprint at bioRxiv. 2020.06.22.160168.

Bobonis, J., Mitosch, K., Mateus, A., Kritikos, G., Elfenbein, J.R., Savitski, M.M., Andrews-Polymenis, H., and Typas, A. (2020a). Phage proteins block and trigger retron toxin/antitoxin systems. Preprint at bioRxiv. 2020.06.22.160242.

Bondy-Denomy, J., Qian, J., Westra, E.R., Buckling, A., Guttman, D.S., Davidson, A.R., and Maxwell, K.L. (2016). Prophages mediate defense against phage infection through diverse mechanisms. ISME J. *10*, 2854–2866.

Calvo-Villamañán, A., Ng, J.W., Planel, R., Ménager, H., Chen, A., Cui, L., and Bikard, D. (2020). On-target activity predictions enable improved CRISPR-dCas9 screens in bacteria. Nucleic Acids Res. *48*, e64.

Chen, I.A., Chu, K., Palaniappan, K., Pillay, M., Ratner, A., Huang, J., Huntemann, M., Varghese, N., White, J.R., Seshadri, R., et al. (2019). IMG/M v.5.0: an integrated data management and comparative analysis system for microbial genomes and microbiomes. Nucleic Acids Res. *47*, D666–D677.

Cheng, R., Huang, F., Wu, H., Lu, X., Yan, Y., Yu, B., Wang, X., and Zhu, B. (2021). A nucleotide-sensing endonuclease from the Gabija bacterial defense system. Nucleic Acids Res. *49*, 5216–5229.

Christie, G.E., and Calendar, R. (2016). Bacteriophage P2. Bacteriophage *6*, e1145782.

Cohen, D., Melamed, S., Millman, A., Shulman, G., Oppenheimer-Shaanan, Y., Kacen, A., Doron, S., Amitai, G., and Sorek, R. (2019). Cyclic GMP-AMP signalling protects bacteria against viral infection. Nature *574*, 691–695.

Court, R., Cook, N., Saikrishnan, K., and Wigley, D. (2007). The crystal structure of λ-gam protein suggests a model for RecBCD inhibition. J. Mol. Biol. *371*, 25–33.

Cui, L., Vigouroux, A., Rousset, F., Varet, H., Khanna, V., and Bikard, D. (2018). A CRISPRi screen in E. coli reveals sequence-specific toxicity of dCas9. Nat. Commun. *9*, 1912.

Cumby, N., Davidson, A.R., and Maxwell, K.L. (2012). The moron comes of age. Bacteriophage *2*, 225–228.

Deatherage, D.E., and Barrick, J.E. (2014). Identification of mutations in laboratory-evolved microbes from next-generation sequencing data using breseq. In Engineering and Analyzing Multicellular Systems (Humana Press), pp. 165–188.

Dedrick, R.M., Jacobs-Sera, D., Guerrero Bustamante, C.A., Garlena, R.A., Mavrich, T.N., Pope, W.H., Cervantes Reyes, J.C., Russell, D.A., Adair, T., Alvey, R., et al. (2017). Prophage-mediated defence against viral attack and viral counter-defence. Nat. Microbiol. *2*, 1–13.

Depardieu, F., Didier, J.P., Bernheim, A., Sherlock, A., Molina, H., Duclos, B., and Bikard, D. (2016). A eukaryotic-like serine/threonine kinase protects staphylococci against phages. Cell Host Microbe *20*, 471–481.

Doron, S., Melamed, S., Ofir, G., Leavitt, A., Lopatina, A., Keren, M., Amitai, G., and Sorek, R. (2018). Systematic discovery of antiphage defense systems in the microbial pangenome. Science *359*, eaar4120.

Duncan-Lowey, B., McNamara-Bordewick, N.K., Tal, N., Sorek, R., and Kranzusch, P.J. (2021). Effector-mediated membrane disruption controls cell death in CBASS antiphage defense. Mol. Cell *81*, 5039–5051.e5.

Dy, R.L., Przybilski, R., Semeijn, K., Salmond, G.P.C., and Fineran, P.C. (2014). A widespread bacteriophage abortive infection system functions through a Type IV toxin-antitoxin mechanism. Nucleic Acids Res. *42*, 4590–4605.

El-Gebali, S., Mistry, J., Bateman, A., Eddy, S.R., Luciani, A., Potter, S.C., Qureshi, M., Richardson, L.J., Salazar, G.A., Smart, A., et al. (2019). The pfam protein families database in 2019. Nucleic Acids Res. *47*, D427–D432.

Engler, C., Kandzia, R., and Marillonnet, S. (2008). A one pot, one step, precision cloning method with high throughput capability. PLoS One *3*, e3647.

Enright, A.J., Iliopoulos, I., Kyrpides, N.C., and Ouzounis, C.A. (1999). Protein interaction maps for complete genomes based on gene fusion events. Nature *402*, 86–90.

Fillol-Salom, A., Miguel-Romero, L., Marina, A., Chen, J., and Penadés, J.R. (2020). Beyond the CRISPR-Cas safeguard: PICI-encoded innate immune

systems protect bacteria from bacteriophage predation. Curr. Opin. Microbiol. *56*, 52–58.

Friedman, D.I., Mozola, C.C., Beeri, K., Ko, C.-C., and Reynolds, J.L. (2011). Activation of a prophage-encoded tyrosine kinase by a heterologous infecting phage results in a self-inflicted abortive infection. Mol. Microbiol. *82*, 567–577.

Galtier, M., De Sordi, L., Maura, D., Arachchi, H., Volant, S., Dillies, M.A., and Debarbieux, L. (2016). Bacteriophages to reduce gut carriage of antibiotic resistant uropathogens with low impact on microbiota composition. Environ. Microbiol. *18*, 2237–2245.

Galtier, M., De Sordi, L., Sivignon, A., de Vallée, A., Maura, D., Neut, C., Rahmouni, O., Wannerberger, K., Darfeuille-Michaud, A., Desreumaux, P., et al. (2017). Bacteriophages targeting adherent invasive Escherichia coli strains as a promising new treatment for Crohn's disease. J. Crohn's Colitis *11*, 840–847.

Gao, L., Altae-Tran, H., Böhning, F., Makarova, K.S., Segel, M., Schmid-Burgk, J.L., Koob, J., Wolf, Y.I., Koonin, E.V., and Zhang, F. (2020). Diverse enzymatic activities mediate antiviral immunity in prokaryotes. Science *369*, 1077–1084.

Ghisotti, D., Finkel, S., Halling, C., Dehò, G., Sironi, G., and Calendar, R. (1990). Nonessential region of bacteriophage P4: DNA sequence, transcription, gene products, and functions. J. Virol. *64*, 24–36.

Gibson, D.G., Young, L., Chuang, R.-Y., Venter, J.C., Hutchison, C.A., and Smith, H.O. (2009). Enzymatic assembly of DNA molecules up to several hundred kilobases. Nat. Methods *6*, 343–345.

Gilchrist, C.L.M., and Chooi, Y.-H. (2021). Clinker & clustermap.js: automatic generation of gene cluster comparison figures. Bioinformatics *37*, 2473–2475.

Goldfarb, T., Sberro, H., Weinstock, E., Cohen, O., Doron, S., Charpak-Amikam, Y., Afik, S., Ofir, G., and Sorek, R. (2015). BREX is a novel phage resistance system widespread in microbial genomes. EMBO J. *34*, 169–183.

Green, R., and Rogers, E.J. (2013). Transformation of chemically competent *E. coli*. In Methods in Enzymology, *529* (Academic Press Inc.), pp. 329–336.

Hatcher, E.L., Zhdanov, S.A., Bao, Y., Blinkova, O., Nawrocki, E.P., Ostapchuck, Y., Schäffer, A.A., and Brister, J.R. (2017). Virus Variation Resource-improved response to emergent viral outbreaks. Nucleic Acids Res. *45*, D482–D490.

Ho, C.H., Wang, H.C., Ko, T.P., Chang, Y.C., and Wang, A.H.J. (2014). The T4 phage DNA mimic protein arn inhibits the DNA binding activity of the bacterial histone-like protein H-NS. J. Biol. Chem. *289*, 27046–27054.

Hussain, F.A., Dubert, J., Elsherbini, J., Murphy, M., VanInsberghe, D., Arevalo, P., Kauffman, K., Rodino-Janeiro, B.K., Gavin, H., Gomez, A., et al. (2021). Rapid evolutionary turnover of mobile genetic elements drives bacterial resistance to phages. Science *374*, 488–492.

Inouye, S., Sunshine, M.G., Six, E.W., and Inouye, M. (1991). Retronphage φR73: an E. coli phage that contains a retroelement and integrates into a tRNA gene. Science *252*, 969–971.

Isaev, A., Drobiazko, A., Sierro, N., Gordeeva, J., Yosef, I., Qimron, U., Ivanov, N.V., and Severinov, K. (2020). Phage T7 DNA mimic protein Ocr is a potent inhibitor of BREX defence. Nucleic Acids Res. *48*, 5397–5406.

Johnson, C.M., Harden, M.M., and Grossman, A.D. (2020). An integrative and conjugative element encodes an abortive infection system to protect host cells from predation by a bacteriophage. Preprint at bioRxiv. 2020.12.13.422588.

Johnson, W.M., and Lior, H. (1988). A new heat-labile cytolethal distending toxin (CLDT) produced by Escherichia coli isolates from clinical material. Microb. Pathog. *4*, 103–113.

Kahn, M., and Helinski, D.R. (1978). Construction of a novel plasmid-phage hybrid: use of the hybrid to demonstrate ColE1 DNA replication *in vivo* in the absence of a ColE1-specified protein. Proc. Natl. Acad. Sci. USA *75*, 2200–2204.

Käll, L., Krogh, A., and Sonnhammer, E.L.L. (2007). Advantages of combined transmembrane topology and signal peptide prediction-the Phobius web server. Nucleic Acids Res. *35*, W429–W432.

Kapfhammer, D., Blass, J., Evers, S., and Reidl, J. (2002). Vibrio cholerae phage K139: complete genome sequence and comparative genomics of related phages. J. Bacteriol. *184*, 6592–6601.

Kimelman, A., Levy, A., Sberro, H., Kidron, S., Leavitt, A., Amitai, G., Yoder-Himes, D.R., Wurtzel, O., Zhu, Y., Rubin, E.M., and Sorek, R. (2012). A vast collection of microbial genes that are toxic to bacteria. Genome Res. *22*, 802–809.

Kita, K., Tsuda, J., Kato, T., Okamoto, K., Yanase, H., and Tanaka, M. (1999). Evidence of horizontal transfer of the EcoO109I restriction-modification gene to Escherichia coli chromosomal DNA. J. Bacteriol. *181*, 6822–6827.

Koonin, E.V., Makarova, K.S., Wolf, Y.I., and Krupovic, M. (2020). Evolutionary entanglement of mobile genetic elements and host defence systems: guns for hire. Nat. Rev. Genet. *21*, 119–131.

Koyuncu, E., Budayeva, H.G., Miteva, Y.V., Ricci, D.P., Silhavy, T.J., Shenk, T., and Cristea, I.M. (2014). Sirtuins are evolutionarily conserved viral restriction factors. mBio *5*, 2249–2263.

Kronheim, S., Daniel-Ivad, M., Duan, Z., Hwang, S., Wong, A.I., Mantel, I., Nodwell, J.R., and Maxwell, K.L. (2018). A chemical defence against phage infection. Nature *564*, 283–286.

Kufer, T.A., Fritz, J.H., and Philpott, D.J. (2005). NACHT-LRR proteins (NLRs) in bacterial infection and immunity. Trends Microbiol. *13*, 381–388.

Kuzmenko, A., Oguienko, A., Esyunina, D., Yudin, D., Petrova, M., Kudinova, A., Maslova, O., Ninova, M., Ryazansky, S., Leach, D., et al. (2020). DNA targeting and interference by a bacterial Argonaute nuclease. Nature *587*, 632–637.

LeGault, K.N., Hays, S.G., Angermeyer, A., McKitterick, A.C., Johura, F.T., Sultana, M., Ahmed, T., Alam, M., and Seed, K.D. (2021). Temporal shifts in antibiotic resistance elements govern phage-pathogen conflicts. Science *373*, eabg2166.

Leipe, D.D., Koonin, E.V., and Aravind, L. (2004). STAND, a class of P-loop NTPases including animal and plant regulators of programmed cell death: multiple, complex domain architectures, unusual phyletic patterns, and evolution by horizontal gene transfer. J. Mol. Biol. *343*, 1–28.

Lindqvist, B.H., Dehò, G., and Calendar, R. (1993). Mechanisms of genome propagation and helper exploitation by satellite phage P4. Microbiol. Rev. *57*, 683–702.

Lopatina, A., Tal, N., and Sorek, R. (2020). Abortive infection: bacterial suicide as an antiviral immune strategy. Annu. Rev. Virol. *7*, 371–384.

Makarova, K.S., Anantharaman, V., Grishin, N.V., Koonin, E.V., and Aravind, L. (2014). CARF and WYL domains: ligand-binding regulators of prokaryotic defense systems. Front. Genet. *5*, 102.

Makarova, K.S., Wolf, Y.I., and Koonin, E.V. (2013). Comparative genomics of defense systems in archaea and bacteria. Nucleic Acids Res. *41*, 4360–4377.

Makarova, K.S., Wolf, Y.I., Snir, S., and Koonin, E.V. (2011). Defense islands in bacterial and archaeal genomes and prediction of novel defense systems. J. Bacteriol. *193*, 6039–6056.

Maura, D., Morello, E., du Merle, L., Bomme, P., Le Bouguénec, C., and Debarbieux, L. (2012). Intestinal colonization by enteroaggregative *Escherichia coli* supports long-term bacteriophage replication in mice. Environ. Microbiol. *14*, 1844–1854.

McGrath, S., Fitzgerald, G.F., and Van Sinderen, D. (2002). Identification and characterization of phage-resistance genes in temperate lactococcal bacteriophages. Mol. Microbiol. *43*, 509–520.

Mestre, M.R., González-Delgado, A., Gutíerrez-Rus, L.I., Martínez-Abarca, F., and Toro, N. (2020). Systematic prediction of genes functionally associated with bacterial retrons and classification of the encoded tripartite systems. Nucleic Acids Res. *48*, 12632–12647.

Millman, A., Bernheim, A., Stokar-Avihail, A., Fedorenko, T., Voichek, M., Leavitt, A., Oppenheimer-Shaanan, Y., and Sorek, R. (2020a). Bacterial retrons function in anti-phage defense. Cell *183*, 1551–1561.e12.

Millman, A., Melamed, S., Amitai, G., and Sorek, R. (2020b). Diversity and classification of cyclic-oligonucleotide-based anti-phage signalling systems. Nat. Microbiol. *5*, 1608–1615.

Montgomery, M.T., Guerrero Bustamante, C.A., Dedrick, R.M., Jacobs-Sera, D., and Hatfull, G.F. (2019). Yet more evidence of collusion: a new viral defense system encoded by *Gordonia* phage carolann. mBio *10*, e02417–18.

CellPress
OPEN ACCESS

Morehouse, B.R., Govande, A.A., Millman, A., Keszei, A.F.A., Lowey, B., Ofir, G., Shao, S., Sorek, R., and Kranzusch, P.J. (2020). STING cyclic dinucleotide sensing originated in bacteria. Nature *586*, 429–433.

Mortier-Barrière, I., Velten, M., Dupaigne, P., Mirouze, N., Piétrement, O., McGovern, S., Fichant, G., Martin, B., Noirot, P., Le Cam, E., et al. (2007). A key presynaptic role in transformation for a widespread bacterial protein: DprA conveys incoming ssDNA to RecA. Cell *130*, 824–836.

Moura de Sousa, J.A., and Rocha, E.P.C. (2022). To catch a hijacker: abundance, evolution and genetic diversity of P4-like bacteriophage satellites. Philos. Trans. R. Soc. Lond. B Biol. Sci. *377*, 20200475.

Myung, H., and Calendar, R. (1995). The old exonuclease of bacteriophage P2. J. Bacteriol. *177*, 497–501.

Nilsson, A.S., Karlsson, J.L., and Haggård-Ljungquist, E. (2004). Site-specific recombination links the evolution of P2-like coliphages and pathogenic enterobacteria. Mol. Biol. Evol. *21*, 1–13.

O'Hara, B.J., Barth, Z.K., McKitterick, A.C., and Seed, K.D. (2017). A highly specific phage defense system is a conserved feature of the Vibrio cholerae mobilome. PLoS Genet. *13*, e1006838.

Oberto, J., Weisberg, R.A., and Gottesman, M.E. (1989). Structure and function of the nun gene and the immunity region of the lambdoid phage HK022. J. Mol. Biol. *207*, 675–693.

Odegrip, R., Nilsson, A.S., and Haggård-Ljungquist, E. (2006). Identification of a gene encoding a functional reverse transcriptase within a highly variable locus in the P2-like coliphages. J. Bacteriol. *188*, 1643–1647.

Ofir, G., Herbst, E., Baroz, M., Cohen, D., Millman, A., Doron, S., Tal, N., Malheiro, D.B.A., Malitsky, S., Amitai, G., and Sorek, R. (2021). Antiviral activity of bacterial TIR domains via immune signalling molecules. Nature *600*, 116–120.

Ofir, G., Melamed, S., Sberro, H., Mukamel, Z., Silverman, S., Yaakov, G., Doron, S., and Sorek, R. (2018). DISARM is a widespread bacterial defence system with broad anti-phage activities. Nat. Microbiol. *3*, 90–98.

Oliveira, P.H., Touchon, M., Cury, J., and Rocha, E.P.C. (2017). The chromosomal organization of horizontal gene transfer in bacteria. Nat. Commun. *8*, 1.

Owen, S.V., Wenner, N., Dulberger, C.L., Rodwell, E.V., Bowers-Barnard, A., Quinones-Olvera, N., Rigden, D.J., Rubin, E.J., Garner, E.C., Baym, M., and Hinton, J.C.D. (2021). Prophages encode phage-defense systems with cognate self-immunity. Cell Host Microbe *29*, 1620–1633.e8.

Pawluk, A., Bondy-Denomy, J., Cheung, V.H.W., Maxwell, K.L., and Davidson, A.R. (2014). A new group of phage anti-CRISPR genes inhibits the type I-E CRISPR-Cas system of *Pseudomonas aeruginosa*. mBio *5*, e00896.

Penner, M., Morad, I., Snyder, L., and Kaufmann, G. (1995). Phage T4-coded Stp: double-edged effector of coupled DNA and tRNA-restriction systems. J. Mol. Biol. *249*, 857–868.

Piel, D., Bruto, M., Labreuche, Y., Blanquart, F., Lepanse, S., James, A., Barcia-Cruz, R., Dubert, J., Lieberman, E., Mathias Wegner, K., et al. (2021). Genetic determinism of phage-bacteria coevolution in natural populations. Preprint at bioRxiv. 2021.05.05.442762.

Pinilla-Redondo, R., Shehreen, S., Marino, N.D., Fagerlund, R.D., Brown, C.M., Sørensen, S.J., Fineran, P.C., and Bondy-Denomy, J. (2020). Discovery of multiple anti-CRISPRs highlights anti-defense gene clustering in mobile genetic elements. Nat. Commun. *11*, 5652.

Rocha, E.P.C., and Bikard, D. (2022). Microbial defenses against mobile genetic elements and viruses: who defends whom from what? PLoS Biol. *20*, e3001514.

Rousset, F., Cabezas-Caballero, J., Piastra-Facon, F., Fernández-Rodríguez, J., Clermont, O., Denamur, E., Rocha, E.P.C., and Bikard, D. (2021). The impact of genetic diversity on gene essentiality within the *Escherichia coli* species. Nat. Microbiol. *6*, 301–312.

Russell, C.W., and Mulvey, M.A. (2015). The extraintestinal pathogenic *Escherichia coli* factor RqlI constrains the genotoxic effects of the RecQ-like helicase RqlH. PLoS Pathog. *11*, e1005317.

Sberro, H., Leavitt, A., Kiro, R., Koh, E., Peleg, Y., Qimron, U., and Sorek, R. (2013). Discovery of functional toxin/antitoxin systems in bacteria by shotgun cloning. Mol. Cell *50*, 136–148.

Schiltz, C.J., Adams, M.C., and Chappie, J.S. (2020). The full-length structure of Thermus scotoductus OLD defines the ATP hydrolysis properties and catalytic mechanism of Class 1 OLD family nucleases. Nucleic Acids Res. *48*, 2762–2776.

Schiltz, C.J., Lee, A., Partlow, E.A., Hosford, C.J., and Chappie, J.S. (2019). Structural characterization of Class 2 OLD family nucleases supports a two-metal catalysis mechanism for cleavage. Nucleic Acids Res. *47*, 9448–9463.

Sharifi, F., and Ye, Y. (2021). Identification and classification of reverse transcriptases in bacterial genomes and metagenomes. Preprint at bioRxiv. 2021.01.26.428298.

Shore, D., Dehò, G., Tsipis, J., and Goldstein, R. (1978). Determination of capsid size by satellite bacteriophage P4. Proc. Natl. Acad. Sci. USA *75*, 400–404.

Snyder, L. (1995). Phage-exclusion enzymes: a bonanza of biochemical and cell biology reagents? Mol. Microbiol. *15*, 415–420.

Steinegger, M., Meier, M., Mirdita, M., Vöhringer, H., Haunsberger, S.J., and Söding, J. (2019). HH-suite3 for fast remote homology detection and deep protein annotation. BMC Bioinformatics *20*, 473.

Steinegger, M., and Söding, J. (2017). MMseqs2 enables sensitive protein sequence searching for the analysis of massive data sets. Nat. Biotechnol. *35*, 1026–1028.

Studier, F.W. (1975). Gene 0.3 of bacteriophage T7 acts to overcome the DNA restriction system of the host. J. Mol. Biol. *94*, 283–295.

Takeda, K., and Akira, S. (2005). Toll-like receptors in innate immunity. Int. Immunol. *17*, 1–14.

Tal, N., Millman, A., Stokar-Avihail, A., Fedorenko, T., Leavitt, A., Melamed, S., Yirmiya, E., Avraham, C., Amitai, G., and Sorek, R. (2021a). Antiviral defense via nucleotide depletion in bacteria. Preprint at bioRxiv. 2021.04.26.441389.

Tal, N., Morehouse, B.R., Millman, A., Stokar-Avihail, A., Avraham, C., Fedorenko, T., Yirmiya, E., Herbst, E., Brandis, A., Mehlman, T., et al. (2021b). Cyclic CMP and cyclic UMP mediate bacterial immunity against phages. Cell *184*, 5728–5739.e16.

Tesson, F., Hervé, A., Touchon, M., d'Humières, C., Cury, J., and Bernheim, A. (2021). Systematic and quantitative view of the antiviral arsenal of prokaryotes. Preprint at bioRxiv. 2021.09.02.458658.

Toro, N., and Nisa-Martínez, R. (2014). Comprehensive phylogenetic analysis of bacterial reverse transcriptases. PLoS One *9*, e114083.

Touchon, M., Bobay, L.M., and Rocha, E.P.C. (2014). The chromosomal accommodation and domestication of mobile genetic elements. Curr. Opin. Microbiol. *22*, 22–29.

Uc-Mass, A., Loeza, E.J., De La Garza, M., Guarneros, G., Hernández-Sánchez, J., and Kameyama, L. (2004). An orthologue of the cor gene is involved in the exclusion of temperate lambdoid phages. Evidence that Cor inactivates FhuA receptor functions. Virology *329*, 425–433.

Xiong, X., Wu, G., Wei, Y., Liu, L., Zhang, Y., Su, R., Jiang, X., Li, M., Gao, H., Tian, X., et al. (2020). SspABCD-SspE is a phosphorothioation-sensing bacterial defence system with broad anti-phage activities. Nat. Microbiol. *5*, 917–928.

Zavilgelsky, G.B., and Kotova, V.Y. (2014). Antirestriction activity of the monomeric and dimeric forms of T7 Ocr. Mol. Biol. *48*, 150–157.

Ziermann, R., and Calendar, R. (1990). Characterization of the cos sites of bacteriophages P2 and P4. Gene *96*, 9–15.

Zimmermann, L., Stephens, A., Nam, S.Z., Rau, D., Kübler, J., Lozajic, M., Gabler, F., Söding, J., Lupas, A.N., and Alva, V. (2018). A completely reimplemented MPI bioinformatics toolkit with a new HHpred server at its core. J. Mol. Biol. *430*, 2237–2243.

STAR★METHODS

KEY RESOURCES TABLE

REAGENT or RESOURCE	SOURCE	IDENTIFIER
**Bacterial and virus strains**		
E. coli K-12 MG1655	Gift of Mazel lab	RefSeq: NC_000913
E. coli C	Collection de l'Institut Pasteur	CIP 104337
E. coli DH10B	Invitrogen	EC0113
E. coli ACE1	Calvo-Villamañán et al., 2020	N/A
Escherichia phage λ	Gift of Luciano Marraffini	RefSeq: NC_001416
Escherichia phage T4	Gift of Laurent Debarbieux	RefSeq: NC_000866
Escherichia phage P1	Gift of Jean-Marc Ghigo	RefSeq: NC_005856
Escherichia phage 186cIts	Gift of Keith Shearwin	RefSeq: NC_001317
Escherichia phage CLB_P2	Maura et al., 2012	N/A
Escherichia phage LF82_P8	Galtier et al., 2017	N/A
Escherichia phage AL505_P2	Galtier et al., 2016	N/A
Escherichia phage T7	Félix d'Hérelle Reference Center for Bacterial Viruses	RefSeq: NC_001604
Escherichia coli bacteriophage P4 sid1	ATCC	ATCC29746-B1
Escherichia phage P2	ATCC	ATCC-29746
Escherichia phage P2_AC1	This study	ENA: SAMEA9990737
Escherichia phage P2_DC1	This study	ENA: SAMEA9990738
Escherichia phage P2_SIDE7	This study	ENA: SAMEA9990739
Escherichia phage P2_SIAC10	This study	ENA: SAMEA9990740
**Chemicals, peptides, and recombinant proteins**		
Kanamycin	Sigma	Cat # K0254
Chloramphenicol	Euromedex	Cat # 3886-C
Carbenicillin	Euromedex	Cat # 1039-A
Anhydrotetracycline hydrochloride	ThermoFisher	Cat # 233131000
Phusion Polymerase	ThermoFisher	Cat # F530L
TURBO DNAse	ThermoFisher	Cat # AM2238
Proteinase K	Eurobio	Cat # GEXPRK01B5
Phenol-chloroform-isoamylalcohol solution	Sigma	Cat # P3803
Chloroform	Sigma	Cat # C2432
**Critical commercial assays**		
NucleoSpin Plasmid	Macherey-Nagel	Cat # 740588.50
**Oligonucleotides**		
All the DNA oligonucleotides are listed in Tables S2A, S2B, and S7B and method details	Eurofins Genomics	N/A
**Recombinant DNA**		
Plasmid psgRNAc	Cui et al., 2018	Addgene Cat #114006
All the plasmids are listed and described in Tables S2A, S2B, and S7B and method details	N/A	N/A
**Software and algorithms**		
Blast+ 2.9.0	NCBI	ftp://ftp.ncbi.nlm.nih.gov/blast/executables/blast+
Python 3.8.5	Python Software Foundation	https://www.python.org/downloads/release/python-380/
MMseqs2	Steinegger and Söding, 2017	https://github.com/soedinglab/MMseqs2
clinker & clustermap.js	Gilchrist and Chooi, 2021	https://github.com/gamcil/clinker
HHpred	Zimmermann et al., 2018	https://toolkit.tuebingen.mpg.de/tools/hhpred

(*Continued on next page*)

Continued

REAGENT or RESOURCE	SOURCE	IDENTIFIER
HH-suite3	Steinegger et al., 2019	https://github.com/soedinglab/hh-suite
Phobius	Käll et al., 2007	https://phobius.sbc.su.se/
MacSyFinder 1.0.2	Abby et al., 2014	https://github.com/gem-pasteur/macsyfinder
SPAdes 3.15.3	Bankevich et al., 2012	http://cab.spbu.ru/software/spades/
Breseq 0.33.2	Deatherage and Barrick, 2014	https://github.com/barricklab/breseq/releases/tag/v0.35.5

RESOURCE AVAILABILITY

Lead contact

Further information and requests for resources and reagents should be directed to and will be fulfilled by the lead contact, David Bikard (david.bikard@pasteur.fr).

Materials availability

All materials generated for this study are available upon request and without restrictions from the lead contact, David Bikard.

All data is available in the main text or the supplemental information.

Data and code availability

- *E. coli* genome sequences used in this study are freely available from RefSeq.
- This paper does not report original code. All programs used to analyze genomes were previously reported and are freely available online (see key resources table).
- Any additional information required to reanalyze the data reported in this paper is available from the lead contact upon request.

EXPERIMENTAL MODEL AND SUBJECT DETAILS

Bacteria

E. coli K-12 MG1655, *E. coli* C or *E. coli* DH10B were grown at 37°C in Lysogeny Broth (LB) medium, unless stated otherwise. Kanamycin (Kan, Sigma), carbenicillin (Carb, Euromedex) and chloramphenicol (Cm, Euromedex) were used at 50 μg/mL, 100 μg/mL and 20 μg/mL respectively.

Phages

Phages were amplified on *E. coli* K-12 MG1655 (λ, T4, P1, 186cIts, CLB_P2, LF82_P8, AL505_P2, T7) or *E. coli* C (P2, AC1, DC1, SIDE7, SIAC10; see also key resources table). Phage stocks were amplified by mixing 100 μL of an overnight culture of *E. coli* with 10 μL of phage stock solution (either pure or diluted) and 5 mL of warm (~50°C) LB + $CaCl_2$ 5 mM + 0.5% agar and poured on LB + $CaCl_2$ 5 mM + 1% agar plates. Plates on which confluent lysis was observed were used to recover the top-agar layer in 1mL of PBS and transfer it to a 50 mL conical tube. The top agar was then disrupted by vortexing until broken into small pieces. Tubes were then left to incubate 10 min at room temperature before centrifugation at 3,000 g for 5 min. Finally, the supernatant was recovered.

P2-like phages AC1, DC1, SIDE7 and SIAC10 were isolated as follows. *E. coli* from Eligo Bioscience's strain collection were grown overnight in deep-well 96-well plates in 1 mL LB medium with agitation and at 37°C. The next day, cultures were diluted 1:100 into 1 mL LB medium and allowed to re-grow at 37°C with agitation for 3 h. The cultures were diluted 1:10 into 1 mL LB medium with or without mitomycin C (Sigma M4287-2MG final concentration of 0.1 μg/mL). After 6 h of incubation with agitation at 37°C, cultures were filtered with a 0.45 μm filter and the lysates were serially diluted and spotted onto lawns of *E. coli C*. Plaques appeared after overnight incubation at 37°C and were reisolated for a total of three times. AC1 and DC1 were obtained from cultures induced with mitomycin while SIDE7 and SIAC10 were obtained from the supernatant of uninduced cultures. Phage DNA was extracted and sequenced as described below. Genomes were assembled using SPAdes 3.15.3 (Bankevich et al., 2012) and deposited with the following accession numbers: ENA: SAMEA9990737 (AC1), ENA: SAMEA9990738 (DC1), ENA: SAMEA9990740 (SIAC10) and ENA: SAMEA9990739 (SIDE7).

METHOD DETAILS

Cloning candidate defense systems

Systems were amplified from the source strains indicated in Tables S2A and S2B, with the exception of 3 systems which were synthesized (Twist Bioscience): the active system from *E. coli* 2862600 as well as two systems from *E. coli* HVH3 and O157:H7 FRIK944

for which we did not detect any activity against our phage panel (Table S2B). All systems were amplified with their native promoters by Phusion PCR (Thermo Fisher) using primers listed in Tables S2A and S2B. We used as vector pFR66, a low-copy plasmid with a pSC101 origin of replication, a kanamycin resistance cassette and a superfolder GFP gene (DNA sequence is provided in Table S7A). pFR66 was made linear by PCR with primers 5'-TTTTGCCTCCTAACTAGGTC-3' and 5'-CCAGGCATCAAATAAAAC GAAAGGCTCAGT-CGAAAGAC-3'. The GFP gene was replaced by each candidate system using the Gibson method (Gibson et al., 2009). All systems were transformed into electrocompetent *E. coli* K-12 MG1655 cells which were prepared as follows: following 100-fold dilution of an overnight culture in 200 mL of LB, cells were grown to OD ~1, harvested (4,000 g - 7 min) and washed three times in ice cold water, before concentration in ~300 μL of 10% glycerol. One microliter of dialyzed Gibson assembly product was transformed into 20 μL of cells. After 1 h of recovery at 37°C in LB medium, transformants were selected on LB + Kan plates. Mutants of PARIS-2 were constructed using the Gibson method (Gibson et al., 2009) after PCR amplification using primers listed in Table S7B. The TIR-NLR and PARIS-2 systems were also cloned on a derivative of pFR66 providing resistance against chloramphenicol (pFD232), giving pFD233 (TIR-NLR) and pFD235 (PARIS-2) respectively. The PARIS-1 system was cloned under the control of an aTc-inducible Ptet promoter on a derivative of pFR66 leading to pFD237. All constructions were verified using Sanger sequencing.

Phage plaque assays

E. coli K-12 MG1655 or *E. coli* C strains carrying each of the systems or the control plasmid pFR66 were grown overnight in LB + Kan. Bacterial lawns were prepared by mixing 250 μL of a stationary culture with 62.5 μL of $CaCl_2$ 1 M and 12.5 mL of LB + 0.5% agar and the mixture was poured onto large square plates (12 x 12 cm) of LB + Kan. Serial dilutions of high-titer (>10^8 pfu/mL) stocks of phages λ, T4, P1, 186cIts (a thermosensitive variant of 186), CLB_P2 (Maura et al., 2012), LF82_P8 (Galtier et al., 2017) and AL505_P2 (Galtier et al., 2016) were spotted on each plate and incubated overnight at 37°C. For phage T7, plates were incubated overnight at room temperature. For the RT-nitrilase + 1TM system, all incubations were performed at room temperature. Information related to our phage panel is provided in key resources table. The next day, plaques were counted and the fold resistance was measured as the number of plaques in the control plate divided by the number of plaques in the presence of each system. When plaques were too small to be counted individually, we considered the most concentrated dilution where no plaque was visible as having a single plaque. The complete list of validated systems is provided in Table S2A, while additional tested systems are provided in Table S2B.

Time course infection experiments

Overnight cultures of K-12 MG1655 cells carrying a control plasmid or a plasmid encoding PARIS from *E. coli* B185 were diluted to OD ~ 0.04 in LB + Kan and arrayed in a 96-well plate. Growth was then monitored in three replicates every 5 min on an Infinite M200Pro (Tecan) at 37°C with shaking. When OD reached ~0.2, cells were either kept uninfected or infected with ~2.10^8 pfus (MOI ~ 5) or ~2.10^5 pfus (MOI ~ 0.005) of phage T7. Growth was then monitored for 6 h post-infection.

Efficiency of centers of infection

To measure the number of infective centers, cells carrying PARIS or a control plasmid (pFR66) were grown in LB + Kan at 37°C to OD ~ 0.4. Cells were infected with T7 phage at a multiplicity of ~0.01 and incubated at 37°C for 5 min. Cells were then harvested (6,000 g – 3 min) and resuspended in LB + Kan to eliminate free phage, and serial dilutions in 100 μL of LB + Kan were prepared. To each dilution, we added 100 μL of phage-sensitive cells (MG1655 + pFR66), 5 mL of LB + 0.5% agar and $CaCl_2$ (5mM final concentration) and the mix was poured onto a LB + Kan plate. Care was taken to make sure the experiment was finished before lysis of infected cells (~20 min post-infection). The next day, infective centers were measured as the number of plaque-forming units on each plate.

Isolation and sequencing of mutant phages

We isolated T7 mutants that overcome PARIS by picking plaques in the lowest dilutions of a spot assay on a lawn of cells carrying PARIS from *E. coli* B185. The resistance phenotype of each mutant was then verified by comparing the number of plaques in the presence or absence of PARIS. To extract phage DNA, 500 μL of high titer stocks (~10^{10} pfu/mL) of wild-type or mutant T7 were treated with TURBO DNAse (Thermo Fisher Scientific) for 30 min at 37°C. DNAse was inactivated with the addition of 5 μL of EDTA 0.5 mM and 25 μL of inactivation reagent at 65°C for 10 min. The supernatant was then treated with 0.5 mg/mL of proteinase K (Eurobio) and SDS 0.5% to release phage DNA from capsids. DNA was then purified as follows: 500 μL of a phenol-chloroform-isoamylalcohol (PCI, 25:24:1) solution (Sigma) were added to the sample which was then vortexed and centrifuged (6,000 g – 5 min). About 500 μL of the upper aqueous phase was transferred to a fresh tube and another 500 μL of PCI solution was added. After vortexing and centrifugation (6,000 g – 5 min), the upper aqueous phase was transferred to a tube containing 500 μL of chloroform. The sample was further vortexed and centrifuged (6,000 g – 5 min), and the upper aqueous phase was transferred to a tube containing 500 μL of cold isopropanol and incubated for 2h at -20°C to precipitate DNA. After centrifugation (16,000 g – 1 min), the DNA pellet was washed with 75% ethanol. Finally, the pellet was air-dried and resuspended in 50 μL of distilled water. Next-generation sequencing was performed using a Nextera XT DNA library preparation kit and the NextSeq 500 sequencing systems (Illumina) at the Mutualized Platform for Microbiology (P2M) at Institut Pasteur. Mutations were identified by mapping sequencing reads to the T7 reference genome using breseq (v. 0.33.2) (Deatherage and Barrick, 2014). Additional T7 mutants

were isolated from *E. coli* MG1655 + PARIS and *E. coli* DH10B + PARIS, the *ocr* gene was amplified using primers 5'-GTACGATG TACCACATGAAACG-3' and 5'-CACTCAGCAGATTCTAAAGCTATTG-3' followed by Sanger sequencing (Table S4B).

Transformation assays

To verify the involvement of Ocr in the activation of PARIS, we cloned either a GFP or the T7 Ocr protein on the pBAD18 vector as follows: using Phusion PCR (ThermoFischer), a GFP fragment was amplified using primers 5'-ACCCGTTTTTTTGGGCTAGCGAATT GATATCCGGAGGCATATCAA-3' and 5'-GCCTTTCGTTTTATTTGATGCCTGGTTTGTAGAGTTCATCCATGC-3' while *ocr* or the *ocr*^{F54V} gene was amplified from T7 or T7^{ocrF54V} genome using primers 5'-GCCTTTCGTTTTATTTGATGCCTGGTTACTCTT-CATC CTCCTCGTACTCC-3' and 5'-ACCCGTTTTTTTGGGCTAGCG-AATTGCAAGGTGCCCTTTA-TGATA-3'. The pBAD18 backbone was amplified using primers 5'-AATTCGCTAGCCCAAAAAAACGG-3' and 5'-CCAGGCATCAAATAAAACGAAAGGCTCAGTCGAAA GAC-3'. Inserts were cloned into the backbone using the Gibson method (Gibson et al., 2009). Constructions were electroporated into K-12 MG1655 cells and validated by Sanger sequencing, before plasmid extraction by miniprep (Macherey-Nagel). Electrocompetent cells of MG1655 carrying PARIS from *E. coli* B185 or a control plasmid were prepared as described above. A hundred nanograms of plasmids were electroporated in both cell types and incubated for 1h at 37°C for recovery. Serial dilutions were then plated on LB agar plates supplemented with 50 μg/mL of kanamycin, 100 μg/mL of carbenicillin and 0.3% of arabinose and CFUs were counted the next day. To investigate EcoKI inhibition by Ocr, we isolated plasmid pET28-Smt3 carrying an EcoKI recognition motif (GCAC[N6]GTT) from *E. coli* MG1655 (*EcoKI*+) and *E. coli* DH10B (*EcoKI*-) to yield methylated and unmethylated DNA respectively. The resulting plasmids were then introduced in MG1655 carrying the above-described plasmids expressing GFP, Ocr or Ocr^{F54V} (induction with 0.3% arabinose) using a rubidium chloride chemical transformation protocol (Green and Rogers, 2013).

CRISPRi-mediated EcoKI knockdown

A sgRNA targeting *hsdR* (5'-ATGTTTTCCGGTGGGCCATT-3') was cloned onto plasmid psgRNAc (Addgene #114006) using the Golden Gate assembly method (Engler et al., 2008), giving pFD236, and introduced into the dCas9-expressing *E. coli* strain ACE1 (Calvo-Villamañán et al., 2020). The fitness of T7 or T7 mutants was then measured in the presence or absence of aTc (0,5 μg/ml), the inducer for dCas9 expression (Figure 2E).

Effect of TIR-NLR on P2-P4 interaction

E. coli C cells carrying either the control plasmid pFD232 or expressing the TIR-NLR system under the control of its native promoter on a low copy vector (pFD233), together with P420 were grown until OD~0.5 in LB + Cm + Kan and infected with P2 at an MOI of ~0.01. The culture was grown for 2 h at 37°C, centrifuged (5 min - 3,000 g) and the supernatant was recovered and filtered. To measure P420 titers, serial dilutions were prepared and 10μl was mixed with 90μl of E. coli C grown to OD~0.8 followed by incubation at room temperature for 30min and plating on LB + Kan. Colony forming units were counted after overnight incubation at 37°C.

P4-Kan construction

P4-Kan is a variant of P4 sid1 (ATCC29746-B1) in which a KanR cassette was introduced in place of the *gop* gene. It was constructed by 3-piece Gibson assembly. The first fragment containing the Kanamycin cassette was made using primers 5'-AATTCGTGGCAT GAGAGAGTTAAAGGATGATTGAACAAGATGGATTGCACGCAGGTTC-3' and 5'-GGATAGTATGAGTAATTTTCAAAATATACTTTCA TATTCAGAAGAACTCGTCAAGAAGGCGATAGAAGGCGATGCGCTGCGA-3' from the pKD4 plasmid (Addgene #45605). The second fragment containing half of the P4 satellite was amplified using 5'-GCTCATGGTGTCGAACGGGCTTTCAG-3' and 5'-CCT TTAACTCT-CTCATGCCACGAATTCTTAAGGATCTTGC-3' from P4 sid1 (ATCC29746-B1) and the third fragment containing the other half of the P4 satellite was amplified using 5'-CTGAAAGCCCGTTCGACACCA-TGAGC-3' and 5'-ATATGAAAGTATATTTT GAAAATTACTCATACTATCCAGCCCTAAGAACACG-3' from the same ATCC source. The assembly was transformed into DH10B cells and selected on LB + 50 μg/mL kanamycin plates.

Competition between P2 and LF82_P8 in the presence of PARIS

E. coli C cells carrying P4-Kan together with either the PARIS system under the control of its natural promoter on a low copy number vector (pFD235) or the control plasmid pFD232, were grown to OD~0.5 and infected with either P2, LF82_P8 or a 1:1 mixture of P2 + LF82_P8 at an MOI of ~0.01. The cultures were incubated for 2h30 at 37°C, centrifuged (5 min - 3,000 g) and the supernatant was recovered and filtered. Titers of P2 were determined by counting plaque forming units in a top-agar overlay of *E. coli C* carrying pFD235. The control experiment with LF82_P8 alone helped ensure that plaques measured in this manner were indeed P2 phage and not mutants of LF82_P8 that escaped defense by PARIS. Titers of LF82_P8 were determined by counting plaque forming units in a top-agar overlay of *E. coli* C lysogenized by P2. The control experiment with P2 alone helped ensure that plaques measured in this manner were indeed LF82_P8 phage and not mutants of P2 that escaped immunity provided by the P2 prophage. In parallel, serial dilutions of the infections were spotted on LB + Cm + Kan agar plates to estimate the number of live bacteria in the culture. The rate of lysogeny during infection with P2 or P2+LF82_P8 in the presence of PARIS was determined by performing a P2-specific PCR on 16 isolated clones for each condition and each replicate of the experiment, using primers 5'-GTGTGTTTAGGTTACTAGATTGACG TACTTATAG-3' and 5'- GGAAGATGAACCGGTAATGAGAGATATCAG-3' (Figure S6).

QUANTIFICATION AND STATISTICAL ANALYSIS

Identification of prophage-encoded systems

We downloaded all 20,125 *E. coli* genomes and encoded protein sequences available on Genbank in August 2020 with any assembly status. We used the presence of the *psu* gene to pinpoint P4-like elements. To extract the systems located between *psu* and *int* genes in these elements, we performed a blastp search (version 2.9.0) against all *E. coli* proteins with an E-value threshold of 10^{-10} using the Psu protein sequence from *E. coli* E101 as a query (WP_000446153.1). For each hit, we searched for the presence of an integrase by keyword (''integrase'') in the 10 downstream genes and retrieved the genes located in between, yielding a total of 5,251 loci after discarding loci that were across two contigs (Table S1A). To reconstruct systems and assess their frequency, all 10,860 proteins from all loci were then clustered using MMseqs2 (version 8ebc9d16b2679eb485803259c8280127801e074b) (Steinegger and Söding, 2017) using a coverage threshold of 60% (-c 0.6 option), resulting in 541 clusters. For each locus, we then defined protein arrangements as a suite of clusters. We identified a total of 318 different protein arrangements. We manually curated the 121 protein arrangements that were present at least 5 times (together accounting for 94.4% of all loci) and grouped together highly similar systems sharing a core set of genes, resulting in 79 systems summarized in Table S1B.

To extract the systems located between gpA and gpQ genes in P2-like prophages, we performed a blastp search against all *E. coli* proteins with an E-value threshold of 10^{-10} using the gpQ protein from Enterophage P2 as a query (NP_046757.1). Similarly as described above, we searched for the presence of gpA by keyword (''replication endonuclease'') in the 10 downstream genes and retrieved the genes located in between as candidate loci, yielding a total of 18,150 loci after discarding loci that were across two contigs. All 66,498 proteins from all loci were then clustered with MMseqs2 (-c 0.6 option), resulting in 846 protein clusters, and protein arrangements were reconstructed as described above, to the exception that we also discarded arrangements that only comprised proteins shorter than 120 amino acids. We identified a total of 1650 different arrangements, including 310 present in at least 10 occurrences (together accounting for 82% of all loci) that were manually curated as described above to yield 169 different systems listed in Table S5B. Selected loci were visualized using clinker and clustermap.js (Gilchrist and Chooi, 2021).

Protein domains were identified using HHpred (Zimmermann et al., 2018) and HH-suite3 (Table S3; Steinegger et al., 2019). Transmembrane domains and signal peptides were identified using Phobius (Käll et al., 2007).

Detection of PARIS

In order to detect two-protein occurrences of the PARIS system (ATPase + DUF4435), we downloaded HMM profiles for the AAA_15 (PF13175), AAA_21 (PF13304) and DUF4435 (PF14491) protein families from the pfam database (El-Gebali et al., 2019). We then used these profiles to detect PARIS with MacSyFinder (v. 1.0.2) (Abby et al., 2014), requiring the two genes to be present (either AAA domain AND DUF4435), with the following score thresholds: 32 (AAA_15), 27 (AAA_21) and 27 (DUF4435) To detect single protein fusions, we browsed the ''domain organisation'' page of the DUF4435 pfam to look for proteins having both AAA_15/AAA_21 and DUF4435 domains. We selected 10 protein sequences with AAA_15+DUF4435 domains and 10 protein sequences with AAA_21+DUF4435 domains and built HMM profiles from these. We then used this profile to detect PARIS with MacSyFinder (v. 1.0.2) (Abby et al., 2014) with the parameter ''loner'', using the following score thresholds: 80 (AAA_15+DUF4435) and 20 (AAA_21+DUF4435). Using these detection rules, we analyzed 21,738 complete genomes retrieved from NCBI RefSeq in May 2021, representing 21 364 and 374 genomes of Bacteria and Archaea respectively.

Identification of hotspots in other bacterial species

From the IMG database (Chen et al., 2019) (accessed on December 1st 2020), we ran the built-in blastp function (e-value 10^{-10}) using the DUF2290 and DUF4435 proteins from *K. pneumoniae* SB5961 and *E. coli* B185 respectively as queries. We then inspected the genomic neighborhood of distant hits using the online interface. We identified candidate hotspots when a neighboring gene frequently occurred next to the DUF2290 or DUF4435 proteins. In this way, we identified the cI gene from *Vibrionales* shown in Figure 7A as well as the peptidase gene from *Baciliales* shown in Figure 7B as potential locations for hotspots. To verify this, we then performed a new blastp search using these genes as queries against the IMG database and inspected the genomic neighborhood of their loci.

Cell Host & Microbe, Volume *30*

Supplemental information

Phages and their satellites encode hotspots of antiviral systems

François Rousset, Florence Depardieu, Solange Miele, Julien Dowding, Anne-Laure Laval, Erica Lieberman, Daniel Garry, Eduardo P.C. Rocha, Aude Bernheim, and David Bikard

Supplementary Figures

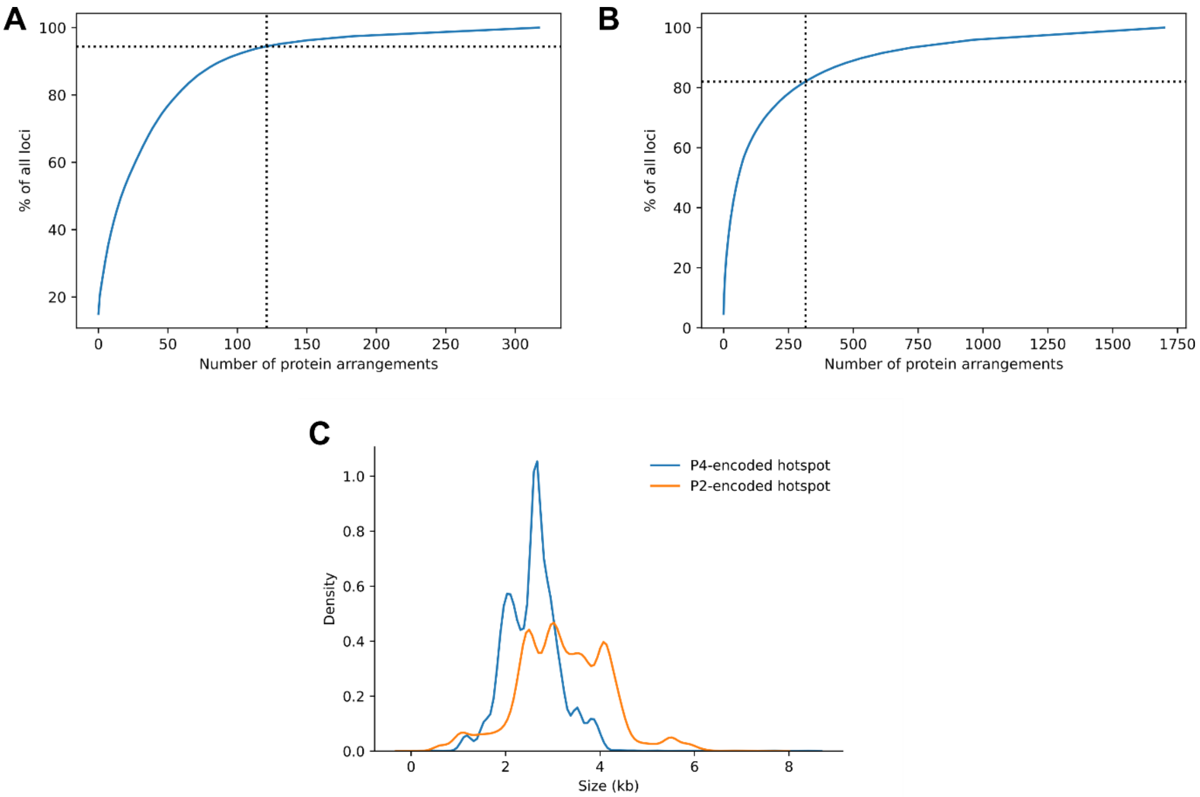

Figure S1. Genomic characteristics of P4 and P2-encoded hotspots, Related to Figure 1 and Figure 4. Plots show the evolution of the frequency of each gene arrangement from P4-encoded (**A**) or P2-encoded (**B**) hotspots. Dashed lines highlight the analyzed threshold: (**A**) 121 arrangements occurring at least 5 times in the P4-encoded hotspot account for 94.4% of all P4-encoded loci, while (**B**) 316 arrangements occurring at least 10 times in the P2-encoded hotspot account for 82% of all P2-encoded loci. (**C**) We measured the size of the P4-encoded hotspot at each locus as the genomic distance between the end of *psu* and the end of *int*. For the P2-encoded hotspot, we used the distance between the end of *A* and the end of *Q*. P2-like phages encode larger systems than P4-like satellites on average (Mann-Whitney p-value $< 10^{-100}$).

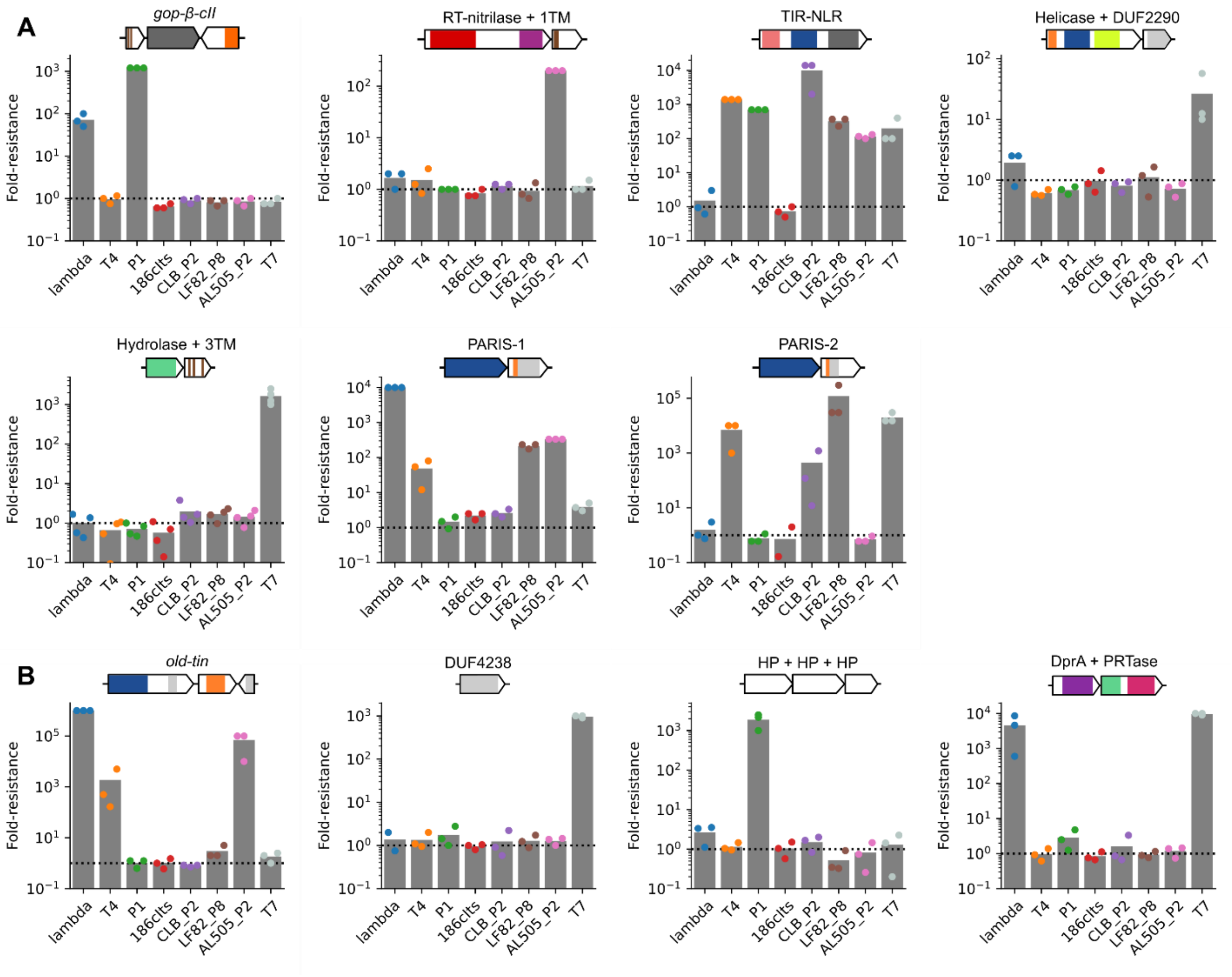

Figure S2. Detailed fold-resistance of all verified defense systems against 8 phages, Related to Figure 2 and Figure 5. Plaque-forming units were measured for each of 8 phages on cells harboring either a control plasmid or a defense system from P4 (**A**) or P2 (**B**) hotspot. Fold-resistance was measured as the ratio between these two values. Bar plots show the mean of 3 to 4 independent measurements. All systems were expressed from their native promoter and measured at 37°C, except the RT-nitrilase + 1TM system that was measured at room temperature and PARIS-1 that was measured in the presence of aTc to induce expression. RT: reverse-transcriptase, TM; transmembrane helix, DUF: domain of unknown function, HP: hypothetical protein, TIR: Toll/Interleukin-1 Receptor, NLR: NOD-like Receptor, PRTase: phosphoribosyltransferase.

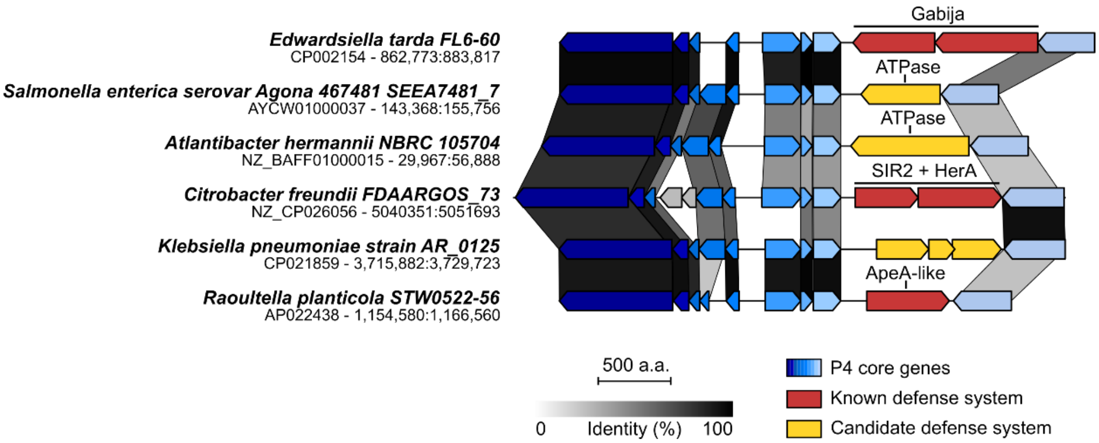

Figure S3. Genetic diversity encoded on P4-like satellites outside *E. coli*, Related to Figure 1. Genomic comparison of P4-like satellites in six different genomes of Enterobacteriaceae. P4 core genes are shown in blue shades. Grey shades show the percentage of identity between homologous proteins from different genomes. Genome accession numbers and positions are shown on the left.

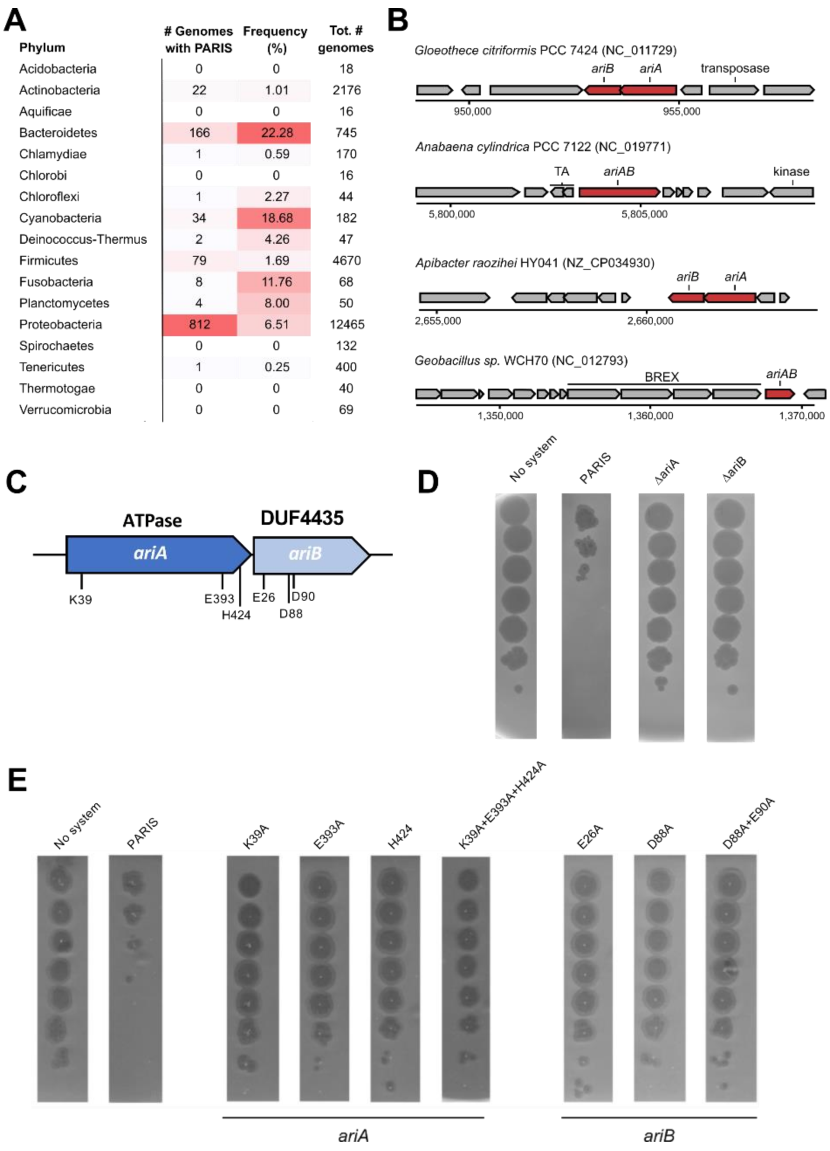

Phylum	# Genomes with PARIS	Frequency (%)	Tot. # genomes
Acidobacteria	0	0	18
Actinobacteria	22	1.01	2176
Aquificae	0	0	16
Bacteroidetes	166	22.28	745
Chlamydiae	1	0.59	170
Chlorobi	0	0	16
Chloroflexi	1	2.27	44
Cyanobacteria	34	18.68	182
Deinococcus-Thermus	2	4.26	47
Firmicutes	79	1.69	4670
Fusobacteria	8	11.76	68
Planctomycetes	4	8.00	50
Proteobacteria	812	6.51	12465
Spirochaetes	0	0	132
Tenericutes	1	0.25	400
Thermotogae	0	0	40
Verrucomicrobia	0	0	69

Figure S4. Detection of PARIS in different prokaryotic phyla and defense phenotype of catalytic site mutants. Related to Figure 3. (**A**) PARIS was searched in 21,738 prokaryotic genomes using HMM searches and MacSyFinder (Tesson et al., 2021). For each clade, cells show the number and proportion of genomes where we detected PARIS. A color gradient is used to depict prevalence. (**B**) Genomic view of a few occurrences of PARIS in bacterial genomes. PARIS occurs either as a two-protein system or as a single protein fusion. PARIS genes are colored in red. Genome accessions are shown in parentheses and the bottom track shows genome coordinates. (**C**) Catalytic residues of PARIS in AriA and AriB were predicted using HHpred (Zimmermann et al., 2018). Resistance against phage T7 was measured for each gene deletion (**D**) and single or multiple amino acid mutants (**E**). Plaque assays are representative of three independent replicates. See also Table S7B.

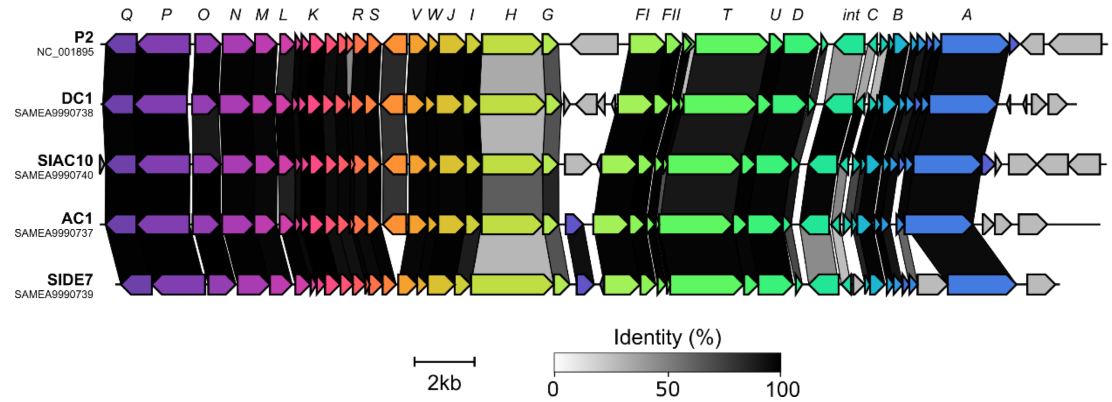

Figure S5. Genomic comparison of canonical phage P2 with newly isolated P2-like phages, Related to Figure 5. Genomes were aligned and visualized using clinker and clustermap.js (Gilchrist and Chooi, 2021). Genes are colored by homologous groups. Grey shades illustrate protein identity.

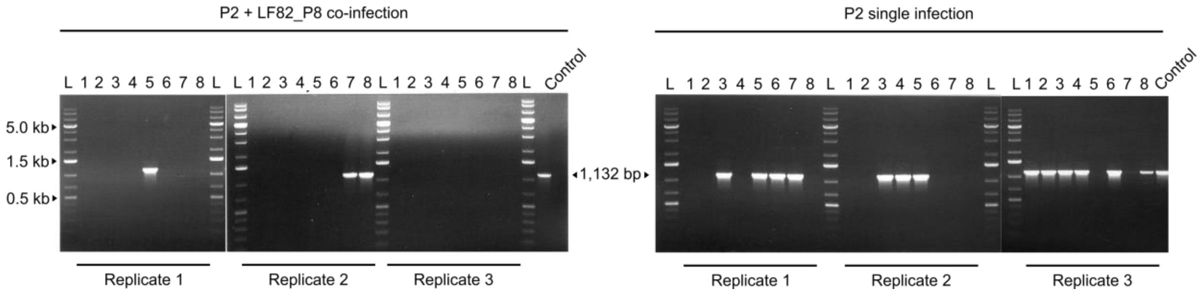

Figure S6. Surviving colonies after co-infection of PARIS-expressing cells by P2 and LF82_P8 include P2 lysogens, Related to Figure 6. E. coli C carrying PARIS (pFD235) and P4-Kan was infected by P2 and LF82_P8 in 1:1 ratio (left) or by P2 alone as a control (right) (see methods). Clones obtained on LB + Cm + Kan plates after infection were screened for the presence of P2 lysogens. A P2-specific PCR was performed on 16 clones for each replicate. The number of positives was 2, 3 and 2, for replicates 1, 2 and 3 respectively after coinfection, and 9, 5 and 13 after infection by P2 alone. The estimated rate of lysogeny is thus ~15% after coinfection and 56% after infection by P2 alone. A P2 lysogen in *E. coli* C was used as a positive control for PCR (see methods). Representative agarose gels for each replicate are shown.

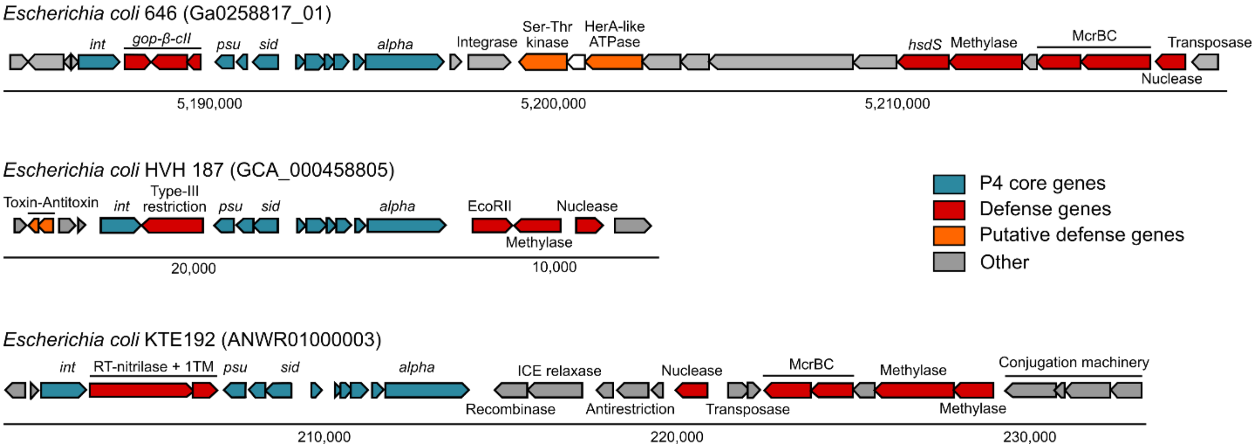

Figure S7. Genomic view of P4-like elements integrated near defense systems and integrative conjugative elements, Related to Figure 1. P4 genes are colored in blue while defense-associated genes are colored in orange or red. Genome accession numbers are shown in parentheses and the bottom tracks shows genomic coordinates.
